# Transcriptional profiling of coaggregation interactions between *Streptococcus gordonii* and *Veillonella parvula* by Dual RNA-Seq

**DOI:** 10.1038/s41598-019-43979-w

**Published:** 2019-05-21

**Authors:** Naresh V. R. Mutha, Waleed K. Mohammed, Natalio Krasnogor, Geok Y. A. Tan, Wei Yee Wee, Yongming Li, Siew Woh Choo, Nicholas S. Jakubovics

**Affiliations:** 1 Department of Biological Sciences, Xi’an Jiaotong-Liverpool University, Suzhou, P.R. China; 2 Institute of Biological Sciences, Faculty of Science, University of Malaya, Kuala Lumpur, Malaysia; 3 School of Dental Sciences, Newcastle University, Newcastle upon Tyne, UK; 4 Department of Basic Science, College of Dentistry, University of Anbar, Ramida, Anbar Iraq; 5 Interdisciplinary Computing and Complex Biosystems (ICOS) Research Group, School of Computing, Newcastle University, Newcastle upon Tyne, UK; 6 School of Science, Monash University Malaysia, Bandar Sunway, Subang Jaya Malaysia; 7 Suzhou Genome Centre (SGC), Health Technologies University Research Centre (HT-URC), Xi’an Jiaotong-Liverpool University, Suzhou Dushu Lake Science and Education Innovation District, Suzhou, China

**Keywords:** Microbial ecology, Biofilms, Applied microbiology, Bacterial transcription

## Abstract

Many oral bacteria form macroscopic clumps known as coaggregates when mixed with a different species. It is thought that these cell-cell interactions are critical for the formation of mixed-species biofilms such as dental plaque. Here, we assessed the impact of coaggregation between two key initial colonizers of dental plaque, *Streptococcus gordonii* and *Veillonella parvula*, on gene expression in each partner. These species were shown to coaggregate in buffer or human saliva. To monitor gene regulation, coaggregates were formed in human saliva and, after 30 minutes, whole-transcriptomes were extracted for sequencing and Dual RNA-Seq analysis. In total, 272 genes were regulated in *V. parvula*, including 39 genes in oxidoreductase processes. In *S. gordonii*, there was a high degree of inter-sample variation. Nevertheless, 69 genes were identified as potentially regulated by coaggregation, including two phosphotransferase system transporters and several other genes involved in carbohydrate metabolism. Overall, these data indicate that responses of *V. parvula* to coaggregation with *S. gordonii* are dominated by oxidative stress-related processes, whereas *S. gordonii* responses are more focussed on carbohydrate metabolism. We hypothesize that these responses may reflect changes in the local microenvironment in biofilms when *S. gordonii* or *V. parvula* immigrate into the system.

## Introduction

It is estimated that approximately 700 different species of bacteria are adapted to colonize the oral cavity of humans^1,2^. The oral cavity of any individual will harbour approximately 100–300 different species of microorganism, which live in biofilms on the surfaces of hard and soft tissues^3^. Biofilm formation is a complex and dynamic process that involves the sequential and ordered colonization of primary and secondary colonizers by selective adherence processes^2^. The processes of adherence and biofilm formation are governed by complex interactions between different bacteria including the exchange of signals and metabolites, and the production of growth-stimulatory or inhibitory compounds^4^. It is hypothesized that mutualistic interactions allow the growth of mixed-species communities under conditions in which neither species could grow alone^5^. *Veillonella* spp. appear to be particularly important in these interactions and can support the growth of model mixed-species communities in human saliva^6^.

*Veillonella* spp. and *Streptococcus* spp. provide an interesting model of cell-cell interactions since they have well-documented synergistic interactions, and they appear to co-occur *in vivo*^7–9^. Oral streptococci are often the most numerous bacteria in early dental plaque and are generally considered commensals in the oral cavity^10,11^. They catabolize carbohydrates to short-chain organic acids, such as lactic acid and pyruvic acid. Previous studies have shown that expression of the gene *amyB*, encoding α-amylase, is up-regulated in *Streptococcus gordonii* DL1 (Challis) cells in close proximity to *V. parvula* PK1910 (previously ‘*V. atypica*’)^12,13^. *Veillonellae* constitute as much as 5% of the initial plaque biomass but are unable to catabolise sugars^14^. They rely on the fermentation of organic acids such as lactic acid that are produced by saccharolytic species such oral streptococci, thus establishing a metabolic food chain in dental plaque^15^. Further, *Veillonella* spp. are juxtaposed with coaggregation receptor polysaccharide-bearing streptococci in early communities *in vivo*, and a rapid succession of *Veillonella* phylotypes occurs in these communities^16^.

The ability of oral streptococci to coaggregate with *Veillonella* species appears to correlate with the colonization site within the oral cavity. Thus, *S. salivarius* is commonly isolated from the tongue and most *S. salivarius* strains coaggregate with *V. atypica* or *V. dispar*, which are also prevalent on the tongue^17^. By contrast, *S. sanguinis* and *S. gordonii* strains do not generally coaggregate with *V. atypica* or *V. dispar* isolated from the tongue, but instead coaggregate with most strains of *V. parvula* isolated from subgingival dental plaque^17^. *S. sanguinis* and *S. gordonii* are more commonly identified in dental plaque than on oral soft tissues^18^. Coaggregation of *V. parvula* (formerly ‘*V. atypica*’) PK1910 with oral streptococci, including *S. gordonii* DL1, is well-documented^19^. A number of *S. gordonii* cell surface proteins have been demonstrated to play roles in coaggregation with different species, including SspA/B, CshA/B and Hsa^8,20,21^. Hsa is particularly important in interactions with *Veillonella* spp., and disruption of the *hsa* gene abrogates interactions with many different *Veillonella* strains^8^. However, an *hsa* knockout strain of *S. gordonii* DL1 retained the ability to coaggregate with *V. parvula* PK1910, indicating that other adhesins are involved in this interaction. *V. parvula* PK1910 is not genetically amenable and no specific genes involved in coaggregation have been identified. Nevertheless, it is possible that coaggregation is mediated by an hemagglutinin protein, since the related strain *Veillonella atypica* OK5 requires the *hag1* gene, encoding a putative haemagglutinin, for coaggregation with *S. gordonii* DL1^22^. These interactions may facilitate metabolic exchange and cell-cell sensing by minimizing the distance between cells in biofilms^23^.

Previous studies have employed microarrays to elucidate gene regulation in *Streptococcus mutans* during the formation of mixed-species biofilms with *V. parvula*^24,25^, but have so far not sought to look at the transcriptomes of both simultaneously. Recently, Dual RNA-Seq has been used to study transcriptional changes in *C. albicans* (fungus) and *S. gordonii* (bacteria) following cell-cell interactions^26,27^. In addition, gene regulation in *S. gordonii* and *Fusobacterium nucleatum* has been monitored during coaggregation in human saliva^28^. In this study we performed transcriptome profiling using a Dual RNA-Seq approach to concurrently identify global changes in *S. gordonii* DL1 and *V. parvula* PK1910 gene expression following coaggregation. This work builds on and improves our understanding of the interactions between *S. gordonii* and *V. parvula* and provides insights into their potential roles during the formation of mixed-species biofilms.

## Results

### Coaggregation between *S. gordonii* and *V. parvula* in human saliva

**Figure 1:**
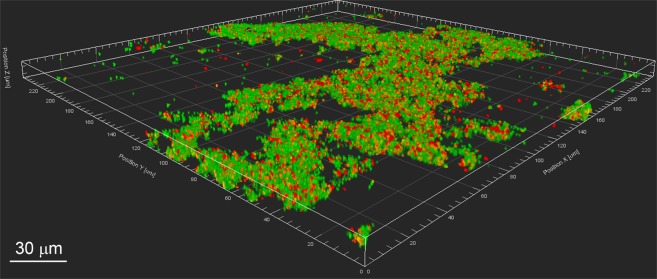
Coaggregation between *S. gordonii* DL1 and *V. parvula* PK1910. Pico-green-stained *S. gordonii* cells (green) and propidium iodide-stained *V. parvula* PK1910 (red) were suspended in human saliva and mixed vigorously to induce coaggregation. Samples were visualized by confocal laser scanning microscopy. A large coaggregated mass is clearly visible, containing *S. gordonii* and *V. parvula* cells interspersed throughout the structure. Bar = 30 µm.

To assess the formation of coaggregates between *S. gordonii* and *V. parvula*, concentrated suspensions of cells in coaggregation buffer were mixed vigorously in as described in ‘Methods’. Within 10 secs, coaggregates were clearly visible and were scored ‘4+’ on the visual scale, indicating large aggregates with almost complete clearing of the background suspension. To ensure that human saliva did not inhibit this interaction, fresh cultures of cells were suspended in freshly collected human saliva, mixed and coaggregation was monitored. Once again, strong coaggregation was observed within seconds and was designated ‘4+’ by reference to the standard visual scoring system. The structure of coaggregates was visualised by pre-staining *S. gordonii* and *V. parvula* with fluorescent dyes and imaging by confocal laser scanning microscopy (CSLM; Fig. 1). Large aggregates, >100 µm in width, were visible and contained *S. gordonii* and *V. parvula* cells evenly mixed throughout the structure.

**Figure 2:**
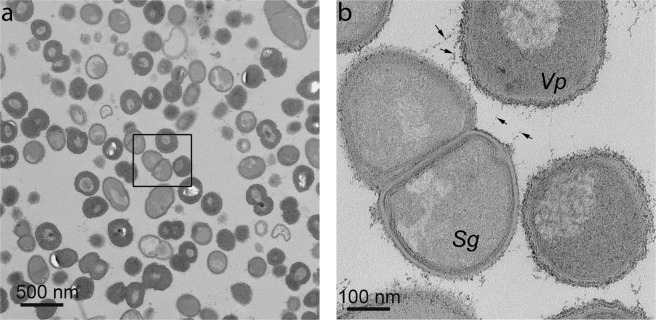
Analysis of coaggregates by TEM. Cells of *S. gordonii* and *V. parvula* were washed, suspended in human saliva and concentrated cultures were mixed vigorously to induce coaggregation. Samples were embedded in resin and visualized by TEM. At relatively low power (**a**), large areas containing densely packed *S. gordonii* and *V. parvula* cells were visible. *S. gordonii* (*Sg*) and *V. parvula* (*Vp*) could be more easily distinguished at higher magnification (**b**). Nearby cells appeared to be connected by extracellular material or fibrils (arrows).

To assess the interactions between *S. gordonii* and *V. parvula* more closely, thin sections through coaggregates were visualized by TEM (Fig. 2). In these images, *S. gordonii* and *V. parvula* cells could be distinguished on the basis of cell wall morphology. Throughout the coaggregate, *S. gordonii* cells were found to be closely associated with *V. parvula* cells (Fig. 2a). At high resolution, there appeared to be structures connecting the cells (Fig. 2b, arrows). Overall, the analysis of coaggregate structure indicated that there was significant potential for cell-cell sensing, as would occur in surface-associated biofilms. Therefore, to assess the impact of coaggregation on gene expression in each organism, monocultures and coaggregate cultures were set up in human saliva. After 30 min, cells were harvested, RNA was extracted and sequenced on the Illumina HiSeq platform.

### Read pre-processing and alignment

A total of 126 million paired end raw reads of 100 bp read length were obtained from nine samples, consisting of three independent experiments for *S. gordonii* monocultures (Sg), *V. parvula* monocultures (Vp) and coaggregate cultures (SgVp). After the removal of low-quality reads, a total of 125 million clean reads (clean ratio = 92.5%) were obtained. After mapping using TopHat, 96% of total raw reads were mapped to Sg monoculture and 43% reads mapped to SgVp_Sg mixed culture samples. For *V. parvula*, 78% of reads mapped to Vp monoculture and 32% reads mapped to SgVp_Vp mixed culture samples as shown in Table S1.

### Transcript abundance and differential expression analysis in mixed and monocultures

To ensure that all samples had a similar range and distribution of expression values, normalization was performed using the Trimmed Mean of M-values (TMM) method. Mixed and monoculture samples after normalization showed a similar distribution of per-gene read counts per sample, as visualized by box plots (Fig. S1). The TMM normalized distributions of data were quantitatively comparable between mixed coaggregate and monoculture samples and no batch effects were apparent from the plots. The distribution of data between samples was further compared by Principal Components Analysis (PCoA). *V. parvula* monoculture and coaggregate samples were clearly separated by the first principal component (Fig. S2). However, only two of the *S. gordonii* coaggregate samples clustered tightly together. The monoculture samples were relatively scattered, indicating poor consistency between samples. Attempts were made to repeat these samples. However, problems with batch effects and contamination made it impossible to interpret the data. Therefore, it was decided to use the three independent repeats for differential expression analysis on the basis that this would provide useful information to form hypotheses for further testing.

**Figure 3:**
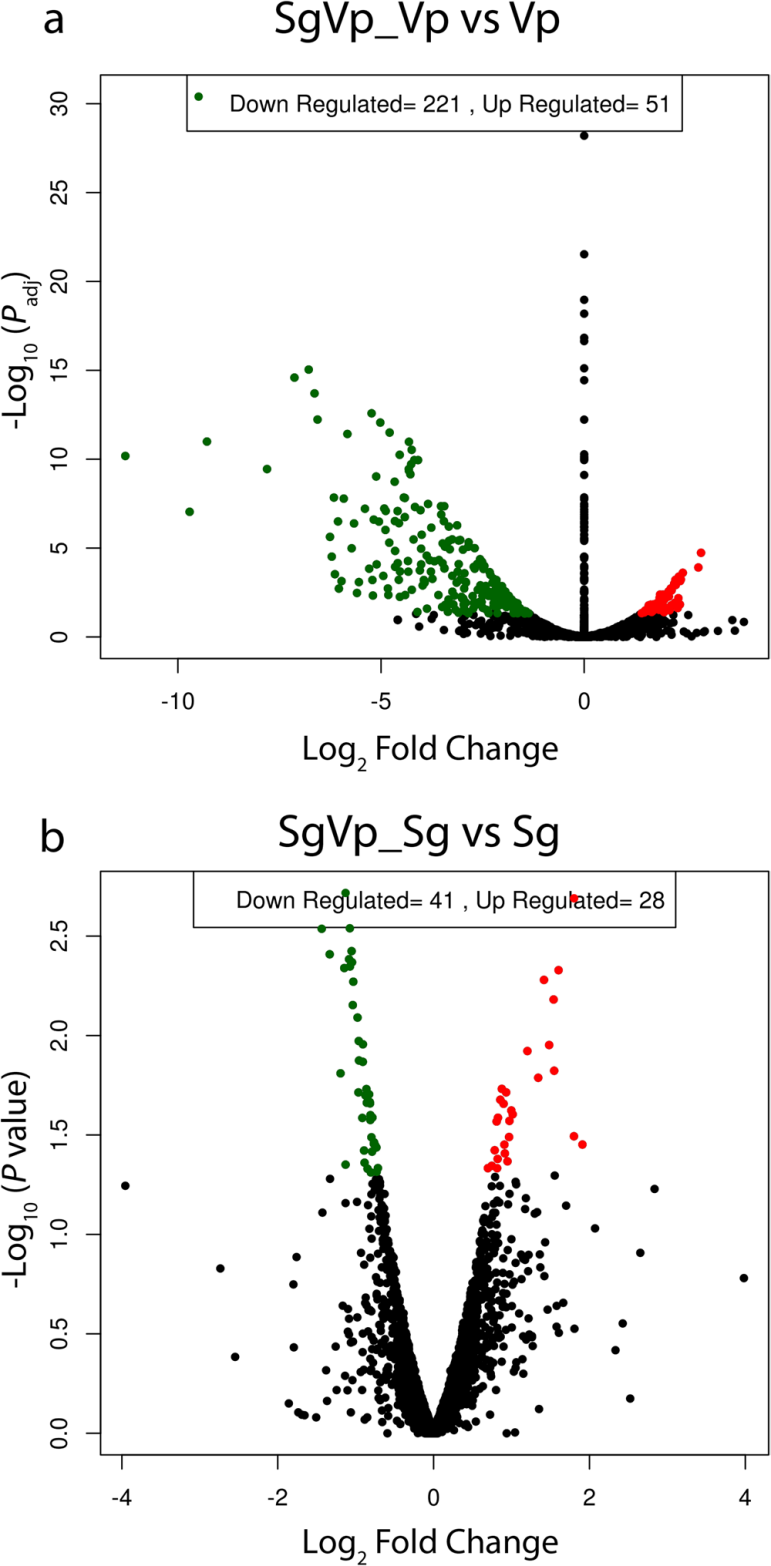
Volcano plots of DESeq results showing gene expression changes. Differentially expressed genes with *P*_adj_ value and a log fold changes are shown as green and red dots. The logarithms of the foldchanges of individual genes (x-axis) are plotted against the negative logarithm of their *P*-value to base 10 (y-axis). Positive log_2_ (fold change) values represent up-regulation and negative values represent down-regulation.

To investigate the impact of coaggregation on gene expression, differential gene expression analysis was performed using DESeq. After comparison between SgVp coaggregate culture and Vp monoculture, a total of 272 significant differentially expressed genes (FDR/*P*_adj_ < 0.05) were obtained (Table S2). An additional 156 genes met the criterion *P* < 0.05 (Table S2). The large number of differentially expressed genes in *V. parvula* suggests that coaggregation has a major impact on gene expression in this species. The differential gene expression was visualized using a volcano plot (Fig. 3a). Differentially expressed genes with positive logarithmic fold change (FC ≥ 1) levels in SgVp compared with Vp were denoted as ‘up-regulated’, while those with negative logarithmic fold change (FC ≤ −1) levels in SgVp were denoted as ‘down-regulated’. Differential expression of *S. gordonii* genes in coaggregate cultures compared with monocultures was assessed using a similar approach. However, none of the *S. gordonii* genes reached the stringent FDR-adjusted significance level *P*_adj_ < 0.05. Therefore, to identify potentially interesting genes, we applied a non-corrected *P* value cut-off of *P* < 0.05. By this measure, 69 significant *S. gordonii* genes were classed as differentially expressed (Fig. 3b, Table S3).

### Validation of RNA-Seq by RT-qPCR

**Figure 4:**
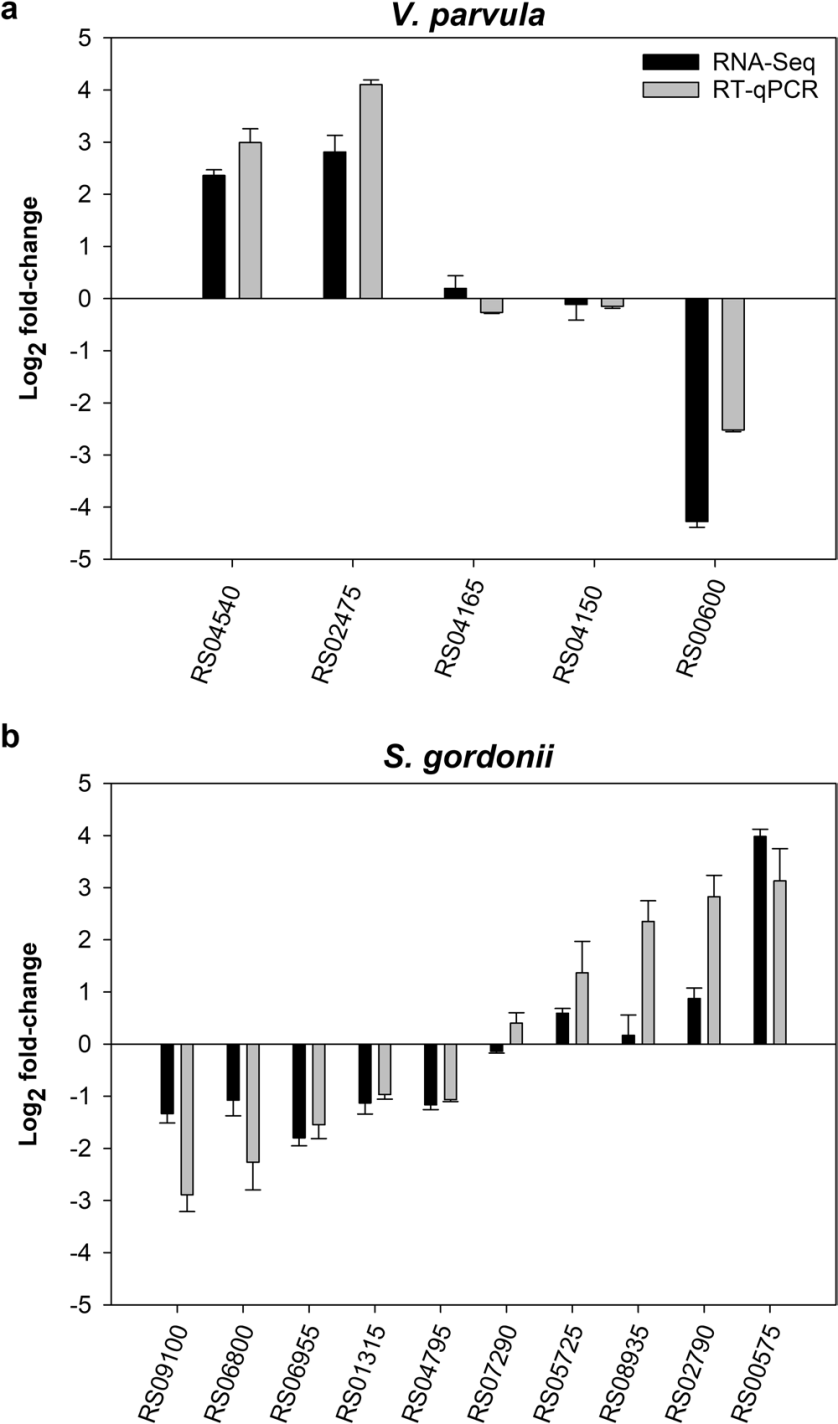
Validation of RNA-Seq data by RT-qPCR. The expression of selected genes in coaggregates versus monocultures was assessed by RT-qPCR, normalized to 16S rRNA expression. Graphs show mean and SD from three independent experiments. In general, there were strong correlations between expression levels measured by RNA-Seq (dark bars) versus RT-qPCR (grey bars).

To confirm that the RNA-Seq was detecting gene regulation appropriately, a selection of genes that were up-regulated or down-regulated were also evaluated by RT-qPCR (Fig. 4). Expression levels were normalised to 16S rRNA expression. Five *V. parvula* genes were selected for analysis including two genes that were strongly up-regulated following coaggregation (RS04540 and RS02475), two genes that were non- significant (RS04165 and RS04150) and one gene that was strongly down-regulated (RS00600). There was a strong correlation between differential expression levels measured by RNA-Seq versus RT-qPCR (Pearson correlation coefficient 0.946, *P* = 0.015), indicating that RNA-Seq was working well for this set of samples. Due to the concerns noted above about the RNA-Seq data for *S. gordonii*, a larger number of *S. gordonii* genes was assessed by RT-qPCR. From ten genes analyzed, there was generally good agreement between RNA-Seq and RT-qPCR. Overall, there was a strong correlation between the data sets from the two different techniques (Pearson correlation coefficient 0.830, *P* = 0.003). Only one gene gave clearly different results with RNA-Seq versus RT-qPCR. This was RS08935, which appeared not regulated by RNA-Seq, but was strongly up-regulated (approximately 5-fold) in coaggregate cultures by RT-qPCR. RNA-Seq also underestimated the gene regulation of several other genes (RS09100, RS06800, RS05725 and RS02790) compared with RT-qPCR. Overall, RNA-Seq detected up- and down-regulated *S. gordonii* genes but appeared to underestimate levels of regulation, which may partially explain why no genes met our originally planned cut-off criterion for differential expression (*P*_adj_ < 0.05). Nevertheless, the data from this analysis provided some justification for applying a less strict cut-off (*P* < 0.05) to identify genes of interest.

### Functional enrichment analysis of *V. parvula* differentially expressed genes between SgVp_Vp and Vp

**Figure 5:**
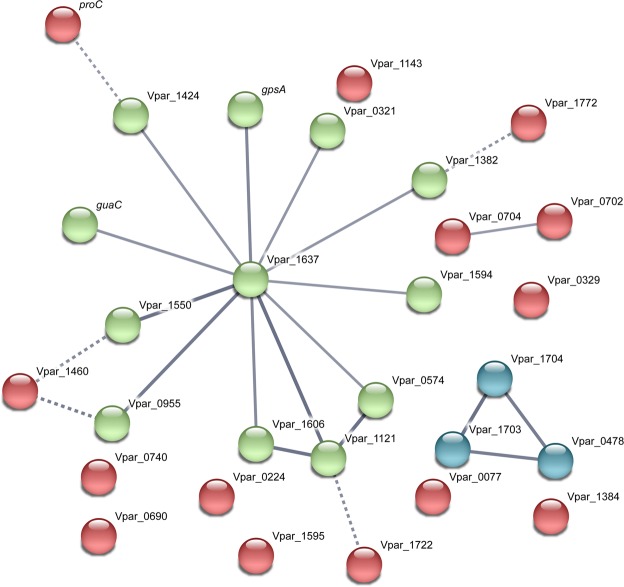
Analysis of interacting gene networks in the ‘oxidoreductase’ category of the *V. parvula* differentially expressed gene dataset. Where possible, genes were assigned names based on the *V. parvula* DSM2008 genome nomenclature by homology searching. The gene list was uploaded to the STRING database, which predicts potential interactions based on factors such as gene environment, protein function or text-mining. The strength of evidence for interactions is indicated by the thickness of edges, with thicker lines indicating stronger interactions. Color coding is based on k-means clustering. The largest network is centered upon Vpar_1637, a predicted glutamate synthase.

To better elucidate the biological functions of differentially expressed genes, we conducted enrichment analysis of Gene Ontology (GO) for the differentially expressed genes by Fisher’s exact test. Only oxidoreductase activity (GO Molecular Function 0016491) and oxidation-reduction process (GO Biological Process 0055114) were significantly enriched in *V. parvula* coaggregate samples compared with monocultures (Table S4). These terms included proteins involved in a broad range of biological processes that involve redox reactions. Interactions between this group of genes were further investigated by analysis with the STRING DB (Fig. 5). Of the 32 genes input into the analysis, 16 genes grouped into one cluster that was centered upon HSIVP1_RS08035 (Vpar_1637, glutamate synthase, downregulated 30-fold in coaggregates). An additional glutamate synthase component, HSIVP1_RS08995, was down-regulated 7.2-fold in coaggregates (Table S4), confirming the importance of this enzyme. Another key glutamate metabolism gene, HSIVP1_RS07610 (Vpar_1550), encoding glutamate dehydrogenase, was strongly down-regulated 53-fold in coaggregates. The STRING DB analysis also identified strong evidence for a connection between HSIVP1_RS08035 and HSIVP1_RS04480 (Vpar_0995), encoding pyruvate dehydrogenase, which was down-regulated 5.7-fold in coaggregates. Similarly, HSIVP1_RS05665 (Vpar_1121), encoding thioredoxin and down-regulated 20-fold in coaggregates, was strongly connected with HSIVP1_RS08035 (glutamate synthase). This gene was also connected with HSIVP1_RS07895 (Vpar_1606; ribonucleoside-diphosphate reductase subunit beta) and HSIVP1_RS02075 (Vpar_0574; dihydrolipoamide dehydrogenase), which were down-regulated 15-fold and 23-fold in coaggregates, respectively. A number of other dehydrogenase and redox-active enzymes were connected to HSIVP1_RS08035 on the basis of weaker evidence.

Two additional gene clusters were identified by STRING DB that were not grouped with HSIVP1_RS08035 glutamate dehydrogenase. One was a pair of genes close to each other on the chromosome, based on the *V. parvula* DSM2008 genome: HSIVP1_RS03260 (Vpar_0702, *fabH*), a component of the fatty acid biosynthesis pathway, and HSIVP1_RS05740 (Vpar_0704, a radical S-adenosyl-L-methionine (SAM) protein, *queE*). Of these, *fabH* was down-regulated 4.1-fold in coaggregates, whereas *queE* was up-regulated 5-fold. The other cluster included two adjacent genes HSIVP1_RS08910 (Vpar_1703, succinate dehydrogenase/fumarate reductase, cytochrome b subunit) and HSIVP1_RS08915 (Vpar_1704, succinate dehydrogenase/fumarate reductase iron-sulfur protein) which were down-regulated 840-fold and 11-fold, respectively. These grouped with HSIVP1_RS02130 (Vpar_0478, cytochrome ubiquinol oxidase subunit I), which was down-regulated 5.9-fold in coaggregates. All three genes encode components of the oxidative phosphorylation pathway.

To investigate functional processes in more detail, gene enrichment analysis was performed using KEGG pathway terms. At a higher level, ‘Metabolic pathways’ (KEGG #1100), ‘Biosynthesis of secondary metabolites’ (KEGG #1110) and ‘Microbial metabolism in diverse environments’ (KEGG #1120) were significantly enriched in the coaggregation-regulated gene set. More specific pathways are listed in Table 1 and included ‘Alanine, aspartate and glutamate metabolism’ (KEGG #250), ‘Arginine and proline metabolism’ (KEGG #330), ‘Mismatch repair’ (KEGG #3430) and ‘Taurine and hypotaurine metabolism (KEGG #430). In general, there was a significant down-regulation of genes involved in multiple amino acid biosynthesis pathways (Fig. S3). Only one gene in these pathways was up-regulated: HSIVP1_RS02475 (VPar_1094, encoding ribose 5-phosphate isomerase B), which was up-regulated 7.0-fold. The gene product interconverts ribulose 5-phosphate and ribose 5-phosphate, which is a substrate for the biosynthesis of histidine. Genes in pathways for biosynthesis of tryptophan, phenylalanine, valine, leucine, isoleucine, threonine, aspartate, glutamate, lysine, arginine and proline were co-ordinately down-regulated in coaggregates, indicating a general reduction in amino acid biosynthesis.

**Table 1:** *V. parvula* genes in key metabolic pathways that were differentially expressed in coaggregates compare with monocultures.

Gene (HSIVP1)	Vpar ID	Gene description	Fold-change^a^	*P*_adj_
*Alanine, aspartate and glutamate metabolism*				
RS00110	Vpar_0075	aspartate aminotransferase	−23	5.5 × 10^−3^
RS01170	Vpar_0278	L-asparaginase	−21	1.8 × 10^−7^
RS01260	Vpar_0296	aspartate racemase	−9.5	2.8 × 10^−4^
RS02565	Vpar_0563	aspartate carbamoyltransferase (*pyrB*)	−3.8	2.8 × 10^−4^
RS07610	Vpar_1550	glutamate dehydrogenase	−53	1.0 × 10^−5^
RS07685	Vpar_1568	argininosuccinate synthase	−11	1.3 × 10^−7^
*Arginine and proline metabolism*				
RS00110	Vpar_0075	aspartate aminotransferase	−23	5.5 × 10^−3^
RS01940	Vpar_0438	s-adenosylmethionine decarboxylase proenzyme	−18	4.9 × 10^−8^
RS06910	Vpar_1429	pyrroline-5-carboxylate reductase (*proC*)	−4.6	6.1 × 10^−3^
RS07610	Vpar_1550	glutamate dehydrogenase	−53	1.0 × 10^−5^
RS07685	Vpar_1568	argininosuccinate synthase	−11	1.3 × 10^−7^
*Mismatch repair*				
RS08370	Vpar_0002	DNA polymerase III	5.1	0.015
RS04315	Vpar_0919	exodeoxyribonuclease small subunit (*xseA*)	−7.8	2.5 × 10^−2^
RS04495	Vpar_0958	DNA polymerase III subunit alpha (*polC*)	3.8	4.7 × 10^−3^
RS05520	Vpar_1000	DNA helicase	2.8	4.2 × 10^−2^
RS05790	Vpar_1213	single-stranded DNA-specific exonuclease	3.1	1.8 × 10^−2^
*Taurine and hypotaurine metabolism*				
RS05910	Vpar_1231	acetate kinase (*ackA*)	−2.7	4.2 × 10^−2^
RS08975	Vpar_1716	phosphate acetyltransferase	−5.7	2.2 × 10^−3^

^a^Fold-change in coaggregates versus monocultures.

Several genes involved in mismatch repair were up-regulated between 3- and 5-fold in coaggregates, including two components of DNA polymerase III and two genes encoding exodeoxyribonucleases (Table 1). Of the genes functioning in mismatch repair, only a DNA helicase was down-regulated in coaggregates. The genes in the taurine/hypotaurine metabolism pathway were Vpar_1231, encoding acetate kinase and Vpar_1716, encoding phosphate acetyltransferase. These genes are both directly involved in the metabolism of acetyl phosphate and were down-regulated in coaggregates. Acetyl phosphate is also a key component of the pyruvate metabolism pathway, and therefore these genes are functionally linked to Vpar_0995, encoding pyruvate dehydrogenase, which was also down-regulated in coaggregates compared with monocultures.

### Functional enrichment analysis of *S. gordonii* differentially expressed genes between SgVp_Sg and Sg

In *S. gordonii*, no statistically significant GO terms met the criteria for being considered enriched (*P*_adj_ < 0.05). From the enrichment analysis, the ‘Biological Processes’ categories with the lowest *P* values were carbohydrate transport (*P* = 1.7 × 10^−2^) and phosphate-containing compound metabolic process (*P* = 2.5 × 10^−2^). Four genes relating to carbohydrate transport were each down-regulated approximately 2-fold in coaggregates. Three of these genes (SGO_RS00210/RS00215/RS00220) encode PTS components EIIB, EIIC and EIID of a mannose, sorbose or fructose transporter, while the other (SGO_RS09095) encodes the EIIBCA components of a sucrose transporter. SGO_RS09095 was also classified under the ‘phosphate-containing compound metabolic process’, along with six other genes. Four of these genes were down-regulated between 1.6- to 1.8-fold and encoded: L-lactate dehydrogenase I *ldh* (SGO_RS06050), a transposase (SGO_RS01075), tagatose-6-phosphate kinase *lacC* (SGO_RS07435), and fructokinase *scrK* (SGO_RS08605). The *dgkA* gene (SGO_RS03505) encoding diacylglycerol kinase was more strongly down-regulated (2.7-fold). The only gene in this category that was up-regulated following coaggregation was *thrB* (SGO_RS03935), encoding homoserine kinase, which was up-regulated 1.9-fold.

**Figure 6:**
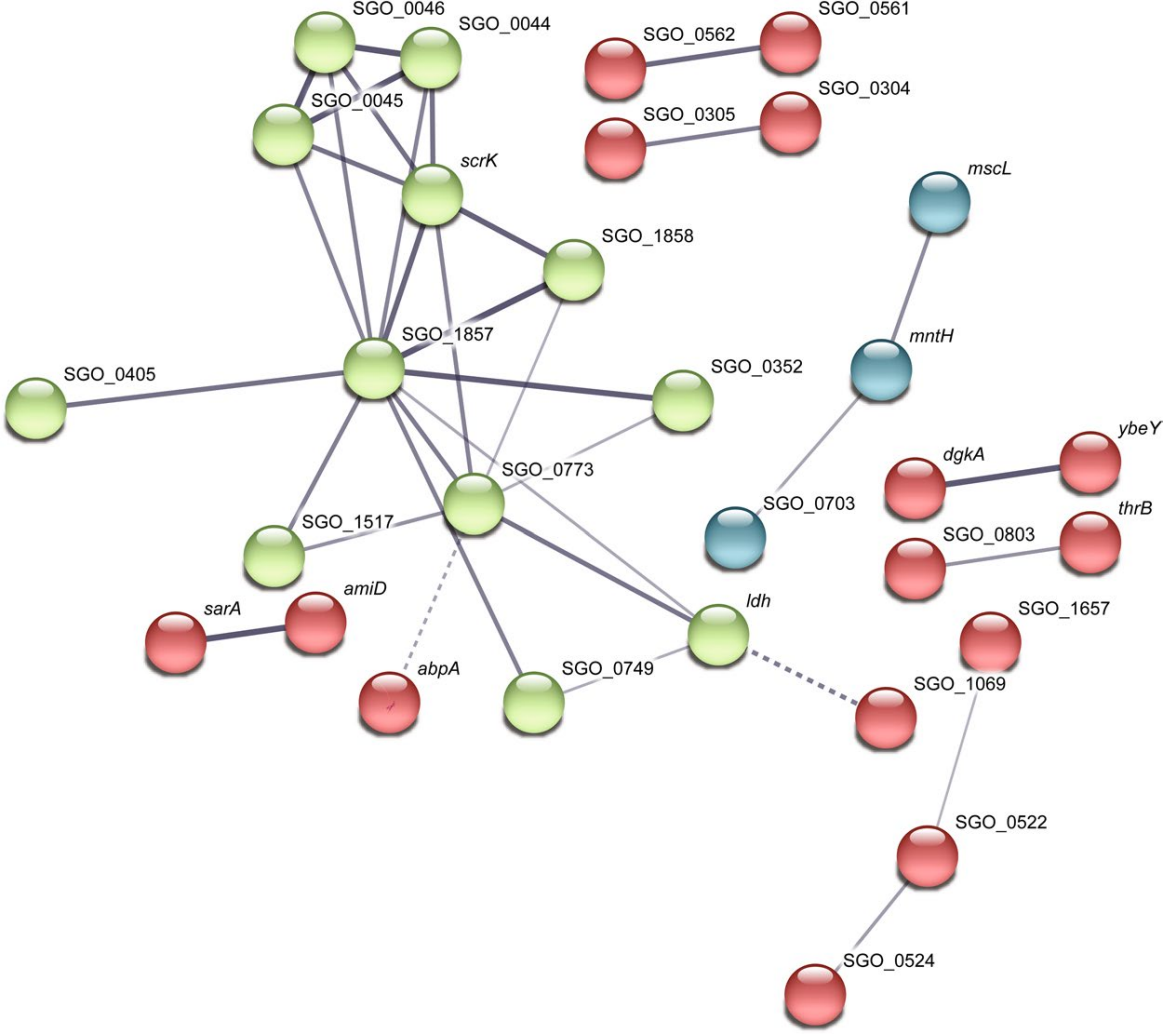
Interacting gene networks in the *S. gordonii* differentially expressed gene dataset, as predicted by STRING DB. Interactions are based on a number of different lines of evidence, with stronger evidence indicated by thicker edges. The coloring of nodes is based on k-means clustering. The network of nodes shown in green is primarily related to carbohydrate metabolism.

A subsequent analysis of gene interactions by STRING DB revealed a large cluster of interacting genes broadly related to carbohydrate transport and metabolism (Fig. 6). These included genes SGO_RS00210/RS00215/RS00220 (old names SGO_0044–0046), *scrK*, *ldh*, *lacC* (SGO_RS07435, old name SGO_1517), SGO_RS09095 (SGO_1857) and a downstream gene SGO_RS09100 (SGO_1858), encoding a putative sucrose-6-phosphate hydrolase, which was down-regulated approximately 2.5-fold in coaggregates. In addition, SGO_RS02020 (SGO_0405), encoding a putative β-n-acetylhexosaminidase, SGO_RS03680 (SGO_0749), encoding glutathione-disulfide reductase, and SGO_RS05250 (SGO_1069), encoding aminopeptidase n, were each up-regulated between 1.8- to 2-fold. The cluster also contained SGO_RS03795 (SGO_0773), encoding carbon catabolite protein A *ccpA*, SGO_RS01740 (SGO_0352), encoding a sugar ABC-type transporter ATP binding protein, and SGO_10300 (*abpA*), encoding an amylase binding protein, which were each down-regulated 1.8- to 1.9-fold in coaggregates.

A number of two- or three-gene interactions were also identified by STRING DB (Fig. 6). The genes *sarA* (*hppA* SGO_RS08395) and *amiD*, (SGO_RS08385), likely encode the ATP binding protein and permease protein, respectively, of a peptide ABC-type transporter. Genes SGO_RS03460 (SGO_0703), encoding a putative Fur family transcriptional regulator designated PerR, SGO_RS01065 (*mntH*), encoding a manganese transporter and SGO_RS06260 (*mscL*), encoding a putative large conductance mechanosensitive channel were linked primarily on the basis of textmining. The *dgkA* gene was linked to the gene immediately upstream, a putative metalloprotease SGO_RS03500 (*ybeY*). Similarly, *thrB* was linked to the adjacent (downstream) gene, SGO_RS03940 (SGO_0803), an uncharacterised protein. Finally, the closely localised genes SGO_RS02580 (SGO_0522) and SGO_RS02590 (SGO_0524), encoding putative acylphosphatase and an hd domain protein (predicted phosphohydrolase enzyme with conserved histidine and/or aspartate residues), respectively, were linked to SGO_RS08120 (SGO_1657), encoding a membrane protein, on the basis of gene co-occurrence and protein homology.

In total, 27 of the 69 regulated *S. gordonii* genes were grouped into networks by STRING DB. Of the remaining 42 genes, two genes were of interest due to their links with carbohydrate metabolism: SGO_RS07610, encoding a putative glucose-1-phosphate adenylyltransferase *glgC*, and SGO_RS08800, encoding pyruvate formate lyase activating enzyme, *pflC*, were each down-regulated 1.7-fold. Two genes, SGO_RS01500 and SGO_RS01505 encode components of a lantibiotic ABC-type transporter permease and were each down-regulated 1.8-fold. The SGO_RS02790 (*sgc*) gene encoding the extracellular serine protease Challisin was up-regulated 1.8-fold, and a Zn-dependent protease encoded by SGO_RS01335 was up-regulated 2-fold. Additional regulated genes encoded ribosomal proteins (two genes), housekeeping functions such as an RNA helicase and esterase, transcriptional regulation or DNA binding (7 genes), stress responses (for example, a universal stress protein), one enzyme in the lysine biosynthesis pathway and several hypothetical, unknown or poorly characterised proteins.

## Discussion

Coaggregation has been suggested to play a key role in promoting interactions between different bacteria that lead to profound phenotypic changes in the partner cells that enable them to proliferate in biofilm formation. Previous studies have shown that cell-cell interactions from coaggregation or biofilm formation lead to changes in gene expression in the partner organisms that may be important for adaptation to a community lifestyle^26,28–30^. Here, *S. gordonii* and *V. parvula* were shown to form 3-dimensional coaggregate structures with cells of different species relatively evenly spread throughout. This is similar to the arrangement of cells that we previously observed in *S. gordonii*-*F. nucleatum* coaggregates^28^. The close proximity of different cell types in these structures may facilitate the exchange of signals or cues that modulate cell-cell sensing and gene regulation. High resolution TEM analysis of these structures revealed close cell-cell interactions with fibrous structures apparently connecting *S. gordonii* and *V. parvula* cells. *S. gordonii* produces fibrils of CshA protein that may be involved in coaggregation^20^. The *V. parvula* cell surface is decorated with pili. However, mutants of *V. parvula* that did not coaggregate with *S. gordonii* still produced pili, indicating that these structures are not essential for coaggregation^19^.

It has previously been shown that cell-cell interactions between *S. gordonii* and *V. parvula* lead to up-regulation of the *S. gordonii amyB* gene, encoding α-amylase, which may mobilise intracellular starch stores to increase production of lactic acid that is a key nutrient for *V. parvula*^12^. This gene regulation is likely mediated by sensing of maltose or malto-oligosaccharides, and is dependent on the central regulator CcpA^13^. The Dual RNA-Seq approach used here has provided a broad overview of the global changes in gene expression in both *S. gordonii* and *V. parvula* following coaggregation. Interestingly, although *S. gordonii amyB* was not specifically found to be regulated following the interaction, a large component of the gene regulation response in this species was centred upon carbohydrate metabolism. By contrast, *V. parvula* exhibited a broad transcriptional response to coaggregation, in particular changes in expression of redox-active functions, down-regulated of amino acid biosynthesis and up-regulation of mismatch repair.

The response of *V. parvula* to coaggregation was consistent with sensing decreased oxidative stress. The most strongly regulated gene in *V. parvula* was HSIVP1_RS09010, encoding superoxide dismutase, which was down-regulated >2,500-fold. This enzyme is a key antioxidant defence in the phylum *Firmicutes*^31^. Two genes encoding thioredoxin were also strongly down-regulated. The thioredoxin system is another important antioxidant defence in bacteria and regulates the activity of many redox-sensitive transcriptional regulators in bacterial cells^32^. It is possible that this system was responsible for driving changes in a wide range of oxidoreductase functions, with diverse metabolic effects on the cells. In addition, HSIVP1_RS09105 encoding catalase was down-regulated 6.6-fold, although it did not quite meet the *P*_adj_ < 0.05 threshold (*P*_adj_ = 0.058, *P* = 0.012). In rich media and over a longer timeframe (6 h), *V. parvula* catalase was up-regulated 180-fold in co-cultures containing *S. gordonii* compared with monocultures^33^. It appears that the short time used here (30 min) was inadequate for *S. gordonii* to generate sufficient levels of H_2_O_2_ to stimulate catalase production by *V. parvula*. In keeping with this hypothesis, the *spxB* gene encoding pyruvate oxidase, the key H_2_O_2_-generating enzyme of *S. gordonii*^34^, was not regulated in response to coaggregation (<1.5-fold change, *P* = 0.37). It is also possible that catalase or peroxidases present in saliva may have inactivated H_2_O_2_. The reduction in expression of key *V. parvula* oxidative stress enzymes is consistent with a model whereby the metabolism of oxygen by *S. gordonii* cells reduces oxidative stress for cells within the coaggregate structure at the early stages of cell-cell interactions. Oxygen gradients are known to occur in microbial biofilms and have been detected in aggregates above a threshold size of radius approximately 35 µm^35^. In addition, obligate and facultative aerobic species including oral streptococci have been shown to protect obligate anaerobes, including veillonellae, and enable them to grow within aerobic biofilms^36,37^.

Interestingly, four genes in the DNA mismatch repair pathway were up-regulated in coaggregates. Oxidative stress causes DNA damage and usually results in increased mismatch repair activity^38^. Therefore, reduction in oxidative stress might be predicted to reduce the need for mismatch repair. It is possible that DNA was protected through a different function of *S. gordonii* cells unrelated to oxygen removal. Alternatively, mismatch repair may also be employed to increase the rate of mutagenesis and facilitate rapid adaptation to a new environment^39^. There was also a strong and co-ordinated down-regulation of amino acid biosynthesis pathways in *V. parvula* in response to coaggregation. It is possible that coaggregation resulted in an increased availability of amino acids in the vicinity of cells. This may occur through the release of amino acids from *S. gordonii* cells. The release of the amino acid ornithine by *S. gordonii* mediates cross-feeding and promotes biofilm development in association with *F. nucleatum*^40^. Alternatively, *V. parvula* may scavenge amino acids from *S. gordonii* cells through the action of an extracellular protease. A similar mechanism has recently been demonstrated in the interaction between *S. gordonii* and *Actinomyces oris*^29^. In this case, the secreted *S. gordonii* protease Challisin appears to scavenge arginine from *A. oris* cells in coaggregates, leading to repression of arginine biosynthesis gene expression in *S. gordonii*. Here, the gene encoding Challisin was up-regulated 1.8-fold following coaggregation, indicating that it may also be important in the interaction with *V. parvula*.

Overall gene regulation in *S. gordonii* appeared much weaker than in *V. parvula*, and no genes were differentially regulated by our planned cut-off value of *P*_adj_ < 0.05. It is possible that *S. gordonii* had a much lower capacity for cell-cell sensing than *V. parvula*. However, we cannot rule out technical issues. For example, Gram-positive streptococcal cells are relatively robust compared with *V. parvula* and it is possible that *S. gordonii* RNA had degraded to some extent prior to RNA-Seq, even though degradation was not apparent on agarose gels or in the BioAnalyzer. It is noteworthy the RT-qPCR generally revealed similar patterns of gene expression as detected by RNA-Seq for both organisms, but several of the *S. gordonii* genes appeared more strongly regulated using RT-qPCR than RNA-Seq. Studies on *S. mutans* have shown stronger levels of gene regulation detected by microarrays than by RNA-Seq.^41,42^. Overall, it appears that RNA-Seq may underestimate streptococcal gene expression for reasons that have yet to be determined. Therefore, for the purpose of gene discovery we elected to use a lower threshold of *P* < 0.05 to consider *S. gordonii* genes potentially regulated. This score resulted in 69 regulated genes, which is broadly in line with previous RNA-Seq studies of *S. gordonii* interactions with *F. nucleatum* or *C. albicans* that identified regulation of 119 *S. gordonii* genes or 72 *S. gordonii* genes, respectively, in response to coaggregation^26,28^.

Although lactate is a key substrate produced by *S. gordonii* that is utilized by *V. parvula*^12^, there was no evidence for the up-regulation of lactate utilization genes in *V. parvula*. Genes involved in lactate utilization including malate L-lactate dehydrogenase (HSIVP1_RS07825), L-lactate dehydrogenase (HSIVP1_RS02235), L-tartrate dehydratase β-subunit (HSIVP1_RS06210) and L-tartrate dehydratase α-subunit (HSIVP1_RS06215) were not regulated (all <1.5-fold change and not significant). These genes were up-regulated in *Veillonella* spp. cultured in saliva from individuals with dental caries compared with saliva from healthy controls, probably as a result of increased lactate availability in caries-associated saliva^43^. However, there is relatively little substrate for lactate production by *S. gordonii* in saliva in the absence of dietary sugar intake. It is likely that lactate did not accumulate to high levels over the 30 min incubation employed here. By contrast, the response of *S. gordonii* to coaggregation was dominated by changes in carbohydrate metabolism and transport genes. Components of a PTS for mannose, sorbose or fructose (SGO_0044–0046) were down-regulated in response to coaggregation. This system has previously been shown to be under the control of the arginine-sensing regulators ArgR and AhrC^44^. However, there was no evidence for a global arginine-sensing response in *S. gordonii* and arginine biosynthesis genes were not regulated in response to coaggregation. Therefore, it is possible that an alternative regulator was acting to control the expression of the PTS genes. More generally, coaggregation led to significant down-regulation in several genes involved in metabolism of sucrose (a PTS transporter and sucrose-6-phosphate hydrolase), fructose (fructokinase, *scrK*), or lactose (*lacC*), uptake of sugar and glycogen biosynthesis (*glgC*). In addition, genes involved in the terminal steps of glucose metabolism, such as lactate dehydrogenase (*ldh*) and pyruvate formate lyase activating enzyme (*pflC*) were also down-regulated. The impact of these changes on central carbon metabolism and on the production of lactic acid as a nutrient source for *V. parvula* is not clear. However, these changes are in agreement with previous studies that have shown a remodelling of carbon metabolism in *S. gordonii* following coaggregation with *V. parvula* by up-regulation of the *amyB* gene^12^.

It is possible that changes in *S. gordonii* carbon metabolism were driven by CcpA, which itself was down-regulated approximately 1.8-fold following coaggregation. CcpA is a master regulator of gene expression in oral streptococci. Previous studies using microarrays and RNA-Seq identified between 45 and 170 genes that were differentially expressed at least two-fold in glucose grown wild-type *S. mutans* cells and an isogenic *ccpA* mutant, including many genes encoding PTS components^41,42^. It is likely that CcpA also plays a broad role in gene regulation in *S. gordonii* in response to extracellular carbon source. In addition to a role in sensing *V. parvula*, *S. gordonii* CcpA controls the expression of the arginine deiminase system, biofilm formation, PTS expression, competence and the peroxidogenic pyruvate oxidase SpxB^45–48^. However, there is evidence that even closely related streptococcal CcpA proteins can have different functions in carbon source sensing^47,48^. Therefore, it will be important to determine the global impact of *ccpA* disruption or down-regulation in *S. gordonii* in future.

## Conclusion

Coaggregation was successfully employed as a model to investigate the impact of cell-cell interactions between *S. gordonii* and *V. parvula* on gene regulation in each species. The major responses of *V. parvula* included down-regulation of genes encoding key proteins involved in oxidative stress resistance, possibly due to a reduction in oxygen tension in the local microenvironment from oxygen metabolism by *S. gordonii*. The transcriptional response of *S. gordonii* was weaker, and was driven by changes in genes associated with carbohydrate metabolism. Further studies investigating the regulation of key genes over time are required to characterize the impact of cell-cell interactions on gene regulation through colonization and biofilm formation on oral tissues.

## Methods

### Saliva preparation

Ethical approval for the collection of saliva from healthy volunteers was obtained from the Newcastle University Research Ethics Committee (reference 1083). All experiments were performed in accordance with the relevant guidelines and regulations. Written informed consent for study participation was obtained from all saliva donors. Parafilm-stimulated saliva was collected from five healthy individuals who had not eaten for at least 2 h prior to collection and was immediately placed on ice. Dithiothreitol (DTT) was added to a final concentration of 2.5 mM and stirred gently on ice for 10 min. Samples were centrifuged at 15,000 g for 30 min at 4 °C to remove large particles. The supernatant was collected and 3 volumes of H_2_O were added to 1 volume of saliva. The diluted saliva was sterilized by filtration through a 0.22 µm pore membrane and aliquots were stored at −20 °C. Before use, saliva was thawed at 37 °C and centrifuged at 1,400 g for 10 min at 20 °C to remove any precipitate.

### Coaggregation assays

*S. gordonii* DL1 was routinely grown statically at 37 °C in THYE broth (30 g/L Bacto™ Todd Hewitt Broth [Difco, Becton Dickinson and Company, Dorcan] 5 g/L yeast extract [Melford Laboratories Ltd.]). *V. parvula* PK1910 was cultured at 37 °C in THYEL consisting of THYE supplemented with 14 mL/L DL-lactate syrup under anaerobic conditions (gas mix 80% N_2_, 10% H_2_ and 10% CO_2_). To visualize coaggregation, cells were cultured for 18 h in THYE or THYEL, harvested by centrifugation at 3,800 g for 10 min and washed three times with one volume of phosphate buffer saline (PBS, pH 7.3). Cells were re-suspended in one volume of coaggregation buffer (1 mM Tris-HCl, pH 8.0, 0.1 mM CaCl_2_, 0.1 mM MgCl_2_, 150 mM NaCl, and 0.02% NaN_3_) and adjusted to OD_600nm_ = 1.0, to give a final concentration of approximately 1 × 10^9^ CFU/mL. To visualize *S. gordonii* cells, an aliquot of 500 µl of cells was added to 5 µl PicoGreen dye (Life technologies Ltd, Paisley, UK) (Invitrogen). For *V. parvula* PK1910, propidium iodide (Sigma-Aldrich, Inc), was added to a concentration of 1.5 mM in 500 µl of bacterial cells. Bacterial suspensions were incubated in darkness at 37 °C for 30 min. Fluorescently stained bacteria were washed twice with coaggregation buffer and resuspended in 500 µl coaggregation buffer. To induce coaggregation in dual-species cultures, 500 µl of each species were mixed by vortex for 10 secs and gently rocked until coaggregation was visible.

To assess gene regulation responses to coaggregation, *S. gordonii* was cultured for 18 h at 37 °C in BHYG containing (per L): Brain Heart Infusion 37 g, Yeast extract 5 g and Na glutamate 2.5 g. *V. parvula* PK1910 was cultured anaerobically at 37 °C in BHYGL consisting of BHYG supplemented with 4 mL/L ≥ 88% DL-lactate syrup and adjusted to pH 7.5 with NaOH before autoclaving. Cells were sub-cultured into fresh medium and grown to mid-exponential phase (OD_600nm_ = 0.4–0.6). Cells were harvested at 3,800 g in a swing out rotor at 20 °C for 10 min and adjusted to OD_600nm_ = 1.0 +/− 0.2. Five mL of each culture was harvested at 3,800 g, 20 °C for 5 min and resuspended in 0.5 ml saliva. Samples were divided into two equal portions and one of each was used for monoculture controls, while the others were vortex mixed for 10 secs to induce coaggregation. All samples were made up to 5 mL by the addition of saliva and incubated at 37 °C for 30 min. Five mL of RNA Later (Invitrogen) was added, the tubes were vortexed for 5 secs and incubated at 20 °C for 5 min. Following incubation, cells were harvested at 3,000 g for 15 min at 20 °C, the supernatant was discarded, and the pellets were frozen at −80 °C for subsequent RNA extraction.

### Coaggregation imaging

Rubber ‘O’ rings of 1 mm thickness (STARLAB, Hamburg, Germany) were fixed to glass microscope slides using sticky wax or superglue. In order to keep the coaggregation stable during imaging, 0.25 volumes of agarose (1.25% wt/vol) were added to the sample. Cells were placed in the middle of the rubber ring and a coverslip (22 × 22 mm) was added. Coaggregation samples were visualized using a fluorescence microscope or a Leica SP2 CLSM microscope (Leica, Microsystems, Heidelberg, Germany) using excitation (Ex) at 530 nm and emission (Em) at 630 nm for propidium iodide and Ex/Em = 485 nm/530 nm for Picogreen.

### RNA extraction

To disrupt cells for RNA extraction, samples were thawed at 20 °C and resuspended in 100 μl spheroplasting buffer containing 0.1 mg/mL spectinomycin^3^. Mutanolysin was added to 500 U/mL and cells were incubated at 37 °C for 5 min. Total RNA was extracted using the Ambion RiboPure Bacteria RNA Purification kit (Life Technologies) according to the manufacturer’s instructions. RNA concentrations were determined using a NanoDrop ND-1000 Spectrophotometer (Thermo Scientific). To ensure that RNA had not degraded during extraction, an aliquot of each sample was analyzed by gel electrophoresis.

### Library preparation and whole-transcriptome sequencing

Library preparation and sequencing were performed at BGI Tech Solutions (Hong Kong). Ribosomal RNA was depleted and remaining mRNA was fragmented. Taking these short fragments as templates, random hexamer primers were used to synthesize the first-strand cDNA. The second-strand cDNA was synthesized using buffer, dNTPs, RNase H and DNA polymerase I, respectively, after removing dNTPs. Short fragments were purified with QiaQuick PCR extraction kit and resuspended in elution buffer for end repair and addition of poly(A) tails. The short fragments were ligated to sequencing adapters. Uracil *N*-glycosylase enzyme was used to degrade the second-strand cDNA, and products were purified by MiniElute PCR Purification Kit before PCR amplification. Finally, the library was sequenced using Illumina HiSeqTM 2500 platform using a paired-end sequencing strategy.

### RNA-Seq data pre-processing and Alignment

Raw reads from Illumina sequencing were obtained in the form of paired-end fastq files. Illumina adapters and low quality reads <Q20 were removed with Trimmomatic-0.36^49^. FastQC^50^ was used to verify removal of low quality reads and adapters. Due to lack of a reference genome for *V. parvula* PK1910, reads were mapped to the closest reference genomes from NCBI, *Veillonella atypica* KON, *Veillonella dispar* ATCC 17748 and *Veillonella parvula* HSIVP1 chromosome using TopHat v1.0.14^51^. This analysis identified *Veillonella parvula* HSIVP1 to be most efficient and accurate for alignment with highest mapping percentage among the three genomes. Reads from three replicates of *S. gordonii* monoculture (Sg) were mapped to the NCBI reference genome (NC_009785.1) using TopHat v1.0.14 with default parameters. Three replicates of mixed samples were mapped separately in two rounds to *S. gordonii* and *V. parvula* and designated ‘SgVp_Sg’ (reads of coaggregate culture mapped to *S. gordonii* reference genome from NCBI) and ‘SgVp_Vp’ (reads of coaggregate culture mapped to *V. parvula*). After read mapping, SAMtools^52^ was employed to calculate mapping statistics.

### Feature quantification, mapping quality assessment and identification of differentially expressed genes

Mapped reads were used for quantification of gene expression using HTseq-count. HTseq.^53^ required a gene feature format (gff) annotation file (mode = union, –t = gene, –i = locus_tag) and the standard gene annotations provided with reference genomes were used. Box plot analysis was used for counts before and after normalization to indicate if the biological replicates were sufficiently similar for subsequent statistical analysis. Comparisons were made between monoculture (Sg, Vp) and coaggregate samples (SgVp_Vp and SgVp_Sg). Differential expression between conditions was determined using the Bioconductor package DESeq version 3.8^54^ in the R statistical software program. DESeq normalized gene count data were based on “size factors” to account for RNA-Seq library size differences and dispersion estimates were calculated. Pairwise comparisons of expression were made between the monoculture and mixed samples group for every replicate based on a negative binomial model. Fold changes were obtained along with their associated *P*-values. The Benjamini-Hochberg method was used to control the false discovery rate (FDR) by adjusting *P*-values to correct for multiple testing. A gene was defined as significantly expressed if it had a Benjamini-Hochberg –adjusted (FDR) *P*-value < 0.05.

### GO classification and enrichment analysis

Gene ontology (GO) annotation analysis was performed on preferentially expressed genes in response to coaggregation using Blast2GO version 5^55^. The nr annotation database was used to obtain the GO term assignments of the transcripts with the Blast2GO program. All significant differentially expressed genes were mapped to each term of the GO database in Blast2GO to calculate the number of genes enriched corresponding to each GO term. Fisher’s exact test was run with default values (a two-tailed test that removes double IDs, with a *P*-value cut-off of 0.05). The final annotation result was categorized with respect to Biological Process, Molecular Function, and Cellular Component.

### Interaction network construction

The STRING version 10.5^56^ database was used to predict if there were any functional associations of differentially regulated significant genes. The search tool for retrieval of interacting genes/proteins (STRING) was used to identify known and predicted interactions (derived from four sources: genomic context, high-throughput experiments, co-expression and previous knowledge). For *V. parvula*, the minimum required interaction score was set to 0.7, whereas this was set to 0.4 for *S. gordonii*. Nodes represent differentially expressed genes and edges indicate the level of confidence in the association, with thicker lines indicating greater confidence. The colors were based on k-means clustering; in each case k-means was set to a value of 3.

### Reverse transcriptase quantitative PCR (RT-qPCR)

Quantitative reverse transcriptase PCR was performed as previously described^28^. Briefly, mRNA was reverse transcribed using the QuantiTect Reverse Transcription Kit (Qiagen, Valencia, CA, USA) with random hexamer primers (Bioline, Taunton, MA, USA). Primers for RT-qPCR analysis are described in Table 2. All RT-qPCR reactions were performed in 25 μL total volume consisting of 0–15 ng template cDNA, forward/reverse primers each at 2 μmol/L, 12.5 μL Power SyBr Green PCR mix (Bioline) and sterile deionized water. Each plate included standard curves consisting of serial 10‐fold dilutions of *S. gordonii* DL1 or *V. parvula* PK1910 chromosomal DNA, along with no template control and ‘no RT’ negative controls. Thermocycling was performed in a DNA Engine Opticon 2 (BioRad, Watford, UK) as follows: (a) 95 °C for 5 minutes; (b) 95 °C for 10 seconds; (c) 60 °C for 30 seconds; (d) plate read; (e) repeat from step ‘b’ a further 39 times; (f) melting curve from 55 to 90 °C, read every 1 °C, hold for 5 seconds. The 16S rRNA gene of *S. gordonii* or *V. parvula* was measured and used to normalize the data. In addition to melting curves, selected products were assessed by agarose gel electrophoresis. Three biological replicates were performed for all RT-qPCR reactions.

**Table 2:** PCR primers used in this study for RT-qPCR analysis of gene expression.

Target locus^a^	Gene product (bp)	Gene description	F/R	Primer sequence (5′-3′)
*V. parvula*				
RS04165	126	Glutamine synthase	F	GGCAATGCATTTGCTAAAGA
			R	AATTGCAACCATGCTACGAA
RS04150	147	Histidine triad protein	F	TGAAGGGTTACTGCCATTTG
			R	GCCCATTTCTTCTCCACCTA
RS04540	89	tRNA µ-54 methyltransferase	F	TCGGTGCGTTTATCCACTAA
			R	ATGCCCGTAGAAATTATGGC
RS02475	89	Sugar family	F	AATGGCCCGTAGAGGATATG
			R	TTTCTCCATCAAATGAAGCG
RS00600	108	Natural resistance-associated macrophage protein (NRAMP)	F	TTGAACAGGCTGAGGAGTTG
			R	TACCTGCCGTAGCAGATGAG
16S *Vp*	108	16S rRNA (*V. parvula*)	F	CCGTGATGGGATGGAAACTGC
			R	CCTTCGCCACTGGTGTTCTTC
*S. gordonii*				
RS09100	95	Sucrose-6-phosphate hydrolase	F	GCCTGATGTGGAGTACCCTT
			R	ATTGGTAGAGCTTGCCGTCT
RS06800	104	Fe-S cluster assembly protein	F	GCCAGACTTTGGACCTGATT
			R	CTTCAGGAACATCATCCCA
RS06955	94	ABC transporter permease	F	TCGAAGTCAAGGGAAGTCCT
			R	ATATAGGCAACCCGATCAGC
RS01315	124	Transporter	F	GCTTGGACCTACTGGGTTGT
			R	TCATTGGCATGGACAGACTT
RS04795	106	Accessory secretory protein Asp5	F	GGTGAAGACCGTCAAATCCT
			R	TCTTACGTGGCTTGAGGTTG
RS07290	108	β-galactosidase	F	GCTATTGCAGCAGATGGAAA
			R	ATGCAATTGGAAACGAACAA
RS05725	127	O family oxidase-dependent iron (Fe^2+^) transporter	F	TTGGCTCATTTATGCTCTGG
			R	TGGATAAATTCCAGCCCAAT
RS08935	96	d-Tyrosyl-tRNA deacylase	F	ACAAGCTCAGGTGTCGATTG
			R	TCATCTGGACCATCATCAGG
RS02790	130	Serine protease (Challisin)	F	ATCCGTCAGAGCAGGAGTCT
			R	TCTCCTGCTGATGGAACTTG
RS00395	118	Hypothetical protein	F	GCTCAAGCATGGCTAACAGA
			R	CTTTAGCACCAGACCAAGCA
16S *Sg*	138	16S rRNA (*S. gordonii*)	F	AGACACGGCCCAGACTCCTAC
			R	TCACACCCGTTCTTCTCTTACAA

^a^‘HSIVP1’ numbers for *V. parvula*; ‘SGO’ numbers for *S. gordonii*.

## Supplementary information

Supplementary Information

## Data Availability

Raw sequence reads were deposited in the NCBI Sequence Reads Archive under BioProject accession number PRJNA505944. All other data generated or analysed during this study are included in this published article (and its Supplementary Information).
