# Supplementary material for: Transcriptional profiling of coaggregation interactions between Streptococcus gordonii and Veillonella parvula by Dual RNA-Seq
Source: Sci Rep. 2019 May 21;9:7664. doi: 10.1038/s41598-019-43979-w (PMC6529473; doi:10.1038/s41598-019-43979-w)
Supplement: Supplementary file 1 — Supplementary Information [file 41598_2019_43979_MOESM1_ESM.pdf]

Supplementary Information for:

Transcriptional profiling of coaggregation interactions between *Streptococcus gordonii* and *Veillonella parvula* by Dual RNA-Seq

Naresh V.R. Mutha, Waleed K. Mohammed, Natalio Krasnogor, Geok Y. A. Tan, Wei Yee Wee, Yongming Li, Siew Woh Choo and Nicholas S. Jakubovics

Contents:

1.. Cover page

2. .... Table S1

3-24. .... Table S2

25-28. .... Table S3

29-30. .... Table S4

31. .... Figure S1

32. .... Figure S2

33. .... Figure S3

34. .... Reference

Table S1. Mapping statistics for assignment of transcripts to species.

Sample	Raw Reads	Cleaned Reads	Reads Mapped	Mapped Reads (% of Total)
S. gordonii	41,753,430	41,530,718	40,180,884	96%
V. parvula	41,514,350	41,292,644	32,368,845	78%
Mixed sample mapped to *S. gordonii* (SgVp_Sg)	42,898,870	42,669,156	18,568,775	43%
Mixed sample mapped to *V. parvula* (SgVp_Vp)	42,898,870	42,669,156	14,056,794	32%

Table S2. Genes regulated in *V. parvula* following coaggregation with *S. gordonii* (P < 0.05).

GeneSymbol (NCBI)	StringDB_locus tags	Gene description	$Log_2FoldChange^a$	P value	P_{adj}^b	GO terms
HSIVP1_RS05385	Vpar_1131	cobalt chelatase	-9.49	0.00	0.00	metal ion binding [GO:0046872]; sirohydrochlorin cobaltochelatase activity [GO:0016852]; anaerobic cobalamin biosynthetic process [GO:0019251]
HSIVP1_RS09425	Vpar_0321	nitroreductase	-6.78	0.00	0.00	oxidoreductase activity [GO:0016491]
HSIVP1_RS07000	- -	NA	-7.13	0.00	0.00	integral component of membrane [GO:0016021]; isomerase activity [GO:0016853]
HSIVP1_RS04385	birA	repressor	-6.64	0.00	0.00	DNA binding [GO:0003677]; serine-type endopeptidase activity [GO:0004252]; DNA repair [GO:0006281]; DNA replication [GO:0006260]; negative regulation of transcription, DNA-templated [GO:0045892]; SOS response [GO:0009432]
HSIVP1_RS00990	- -	C4-dicarboxylate ABC transporter	-5.23	0.00	0.00	integral component of membrane [GO:0016021]; C4-dicarboxylate transmembrane transporter activity [GO:0015556]
HSIVP1_RS00195	Vpar_0077	fad dependent oxidoreductase	-6.56	0.00	0.00	FAD binding [GO:0071949]; oxidoreductase activity [GO:0016491]
HSIVP1_RS05180	- -	sugar-binding protein	-5.02	0.00	0.00	carbohydrate binding [GO:0030246]
HSIVP1_RS01615	menB	naphthoate synthase	-4.79	0.00	0.00	1,4-dihydroxy-2-naphthoyl-CoA synthase activity [GO:0008935]; menaquinone biosynthetic process [GO:0009234]
HSIVP1_RS05950	- -	conserved domain protein	-5.83	0.00	0.00	integral component of membrane [GO:0016021]
HSIVP1_RS03580	Vpar_0002	dna polymerase i	-9.28	0.00	0.00	DNA binding [GO:0003677]; DNA-directed DNA polymerase activity [GO:0003887]; nuclease activity [GO:0004518]; DNA repair [GO:0006281]; DNA-dependent DNA replication [GO:0006261]
HSIVP1_RS05335	rpsH	binding domain protein	-4.31	0.00	0.00	cofactor binding [GO:0048037]
HSIVP1_RS02070	- -	peptidase propeptide and ypeb domain protein	-4.25	0.00	0.00	0
HSIVP1_RS02075	- -	dihydrolipoamide dehydrogenase	-4.54	0.00	0.00	cell [GO:0005623]; dihydrolipoyl dehydrogenase activity [GO:0004148]; electron transfer activity [GO:0009055]; flavin adenine dinucleotide binding [GO:0050660]; cell redox homeostasis [GO:0045454]
HSIVP1_RS09010	Vpar_1722	superoxide dismutase	-11.29	0.00	0.00	metal ion binding [GO:0046872]; superoxide dismutase activity [GO:0004784]
HSIVP1_RS01075	- -	autonomous glycyl radical cofactor	-4.18	0.00	0.00	formate C-acetyltransferase activity [GO:0008861]; lyase activity [GO:0016829]
HSIVP1_RS01885	- -	late embryogenesis abundant protein	-4.09	0.00	0.00	0
HSIVP1_RS00835	- -	family domain protein	-4.26	0.00	0.00	0
HSIVP1_RS02420	- -	lipid a biosynthesis 2- -lipid iva acyltransferase	-4.32	0.00	0.00	#N/A
HSIVP1_RS07925	aroC	chorismate synthase	-7.81	0.00	0.00	#N/A
HSIVP1_RS02560	Vpar_1828	atpase	-4.31	0.00	0.00	#N/A
HSIVP1_RS00600	Vpar_0145	natural resistance-associated macrophage protein	-4.28	0.00	0.00	integral component of membrane [GO:0016021]; plasma membrane [GO:0005886]; metal ion transmembrane transporter activity [GO:0046873]
HSIVP1_RS08175	sepF	hypothetical protein	-5.12	0.00	0.00	0
HSIVP1_RS09185	- -	pf12889 family protein	-4.66	0.00	0.00	0

GeneSymbol (NCBI)	StringDB_locus tags	Gene description	$Log_2FoldChange^a$	*P* value	P_{adj}^b	GO terms
HSIVP1_RS01255	glmM	phosphoglucosamine mutase	-4.44	0.00	0.00	magnesium ion binding [GO:0000287]; phosphoglucosamine mutase activity [GO:0008966]; carbohydrate metabolic process [GO:0005975]
HSIVP1_RS05425	cobD	cobalamin biosynthesis protein	-6.16	0.00	0.00	cobalamin biosynthetic process [GO:0009236]
HSIVP1_RS04860	- -	sufd domain protein	-4.41	0.00	0.00	iron-sulfur cluster assembly [GO:0016226]
HSIVP1_RS07880	Vpar_1857	membrane protein	-5.92	0.00	0.00	integral component of membrane [GO:0016021]
HSIVP1_RS01385	- -	nad h-dependent oxidoreductase	-3.84	0.00	0.00	6,7-dihydropteridine reductase activity [GO:0004155]
HSIVP1_RS05955	- -	glutaconyl- decarboxylase subunit beta	-3.52	0.00	0.00	integral component of membrane [GO:0016021]; plasma membrane [GO:0005886]; glutaconyl-CoA decarboxylase activity [GO:0018801]; sodium ion transport [GO:0006814]
HSIVP1_RS07510	sepF	hypothetical protein	-3.44	0.00	0.00	0
HSIVP1_RS01940	speD	s-adenosylmethionine decarboxylase proenzyme	-4.16	0.00	0.00	#N/A
HSIVP1_RS08035	Vpar_1637	glutamate synthase	-4.92	0.00	0.00	glutamate synthase (NADPH) activity [GO:0004355]; glutamate biosynthetic process [GO:0006537]
HSIVP1_RS06885	- -	uroporphyrinogen-iii c-methyltransferase	-5.40	0.00	0.00	NAD binding [GO:0051287]; precorrin-2 dehydrogenase activity [GO:0043115]; sirohydrochlorin ferrochelatase activity [GO:0051266]; uroporphyrin-III C-methyltransferase activity [GO:0004851]; uroporphyrinogen-III synthase activity [GO:0004852]; cobalamin biosynthetic process [GO:0009236]; siroheme biosynthetic process [GO:0019354]
HSIVP1_RS06315	- -	glutaredoxin	-4.03	0.00	0.00	0
HSIVP1_RS00020	- -	m48 family	-4.88	0.00	0.00	metalloendopeptidase activity [GO:0004222]
HSIVP1_RS02135	Vpar_0478	cytochrome d ubiquinol subunit ii	-4.59	0.00	0.00	integral component of membrane [GO:0016021]; oxidoreductase activity [GO:0016491]
HSIVP1_RS08910	- -	heterodisulfide reductase subunit b	-9.71	0.00	0.00	0
HSIVP1_RS07685	argG	argininosuccinate synthase	-3.52	0.00	0.00	cytoplasm [GO:0005737]; argininosuccinate synthase activity [GO:0004055]; ATP binding [GO:0005524]; arginine biosynthetic process [GO:0006526]
HSIVP1_RS01170	Vpar_0278	l- type ii	-4.41	0.00	0.00	asparaginase activity [GO:0004067]; asparagine metabolic process [GO:0006528]
HSIVP1_RS09140	- -	caax amino protease	-5.18	0.00	0.00	integral component of membrane [GO:0016021]
HSIVP1_RS07575	- -	pf05167 family	-4.66	0.00	0.00	0
HSIVP1_RS07535	atpH	atp synthase delta subunit	-3.45	0.00	0.00	plasma membrane [GO:0005886]; proton-transporting ATP synthase complex, catalytic core F(1) [GO:0045261]; proton-transporting ATP synthase activity, rotational mechanism [GO:0046933]; ATP synthesis coupled proton transport [GO:0015986]
HSIVP1_RS04625	Vpar_1857	membrane protein	-6.06	0.00	0.00	integral component of membrane [GO:0016021]
HSIVP1_RS05410	Vpar_1139	precorrin-8x methylmutase	-5.05	0.00	0.00	precorrin-8X methylmutase activity [GO:0016993]; cobalamin biosynthetic process [GO:0009236]
HSIVP1_RS01750	pyrF	orotidine 5 -phosphate decarboxylase	-4.56	0.00	0.00	orotidine-5'-phosphate decarboxylase activity [GO:0004590]; 'de novo' pyrimidine nucleobase biosynthetic process [GO:0006207]; 'de novo' UMP biosynthetic process [GO:0044205]
HSIVP1_RS06330	Vpar_0835	methyltransferase	-5.66	0.00	0.00	0
HSIVP1_RS09435	whiA	sporulation protein	-3.13	0.00	0.00	0

GeneSymbol (NCBI)	StringDB_locus tags	Gene description	$Log_2FoldChange$[a]	*P* value	P_{adj}[b]	GO terms
HSIVP1_RS07585	- -	phosphoglucomutase	-3.33	0.00	0.00	magnesium ion binding [GO:0000287]; phosphoglucomutase activity [GO:0004614]; carbohydrate metabolic process [GO:0005975]
HSIVP1_RS07820	Vpar_1594	molybdopterin oxidoreductase	-3.76	0.00	0.00	electron transfer activity [GO:0009055]; iron-sulfur cluster binding [GO:0051536]; metal ion binding [GO:0046872]; molybdopterin cofactor binding [GO:0043546]
HSIVP1_RS00960	Vpar_0076	transcriptional family	-4.89	0.00	0.00	DNA binding [GO:0003677]; DNA-binding transcription factor activity [GO:0003700]; transcription, DNA-templated [GO:0006351]
HSIVP1_RS04395	- -	site-specific phage integrase family	-4.00	0.00	0.00	DNA binding [GO:0003677]; DNA integration [GO:0015074]; DNA recombination [GO:0006310]
HSIVP1_RS03615	Vpar_1500	ribosomal protein l7ae	-6.26	0.00	0.00	ribosome [GO:0005840]; RNA binding [GO:0003723]
HSIVP1_RS02525	Vpar_0554	signal peptidase i	-3.25	0.00	0.00	integral component of membrane [GO:0016021]; serine-type peptidase activity [GO:0008236]
HSIVP1_RS00595	sepF	hypothetical protein	-4.20	0.00	0.00	0
HSIVP1_RS04795	gltX	glutamate--trna ligase	-3.05	0.00	0.00	cytoplasm [GO:0005737]; ATP binding [GO:0005524]; glutamate-tRNA ligase activity [GO:0004818]; tRNA binding [GO:0000049]; zinc ion binding [GO:0008270]; glutamyl-tRNA aminoacylation [GO:0006424]
HSIVP1_RS01485	sepF	hypothetical protein	-3.09	0.00	0.00	integral component of membrane [GO:0016021]
HSIVP1_RS03190	Vpar_0685	capsular polysaccharide biosynthesis protein	-3.33	0.00	0.00	manganese ion binding [GO:0030145]; protein tyrosine phosphatase activity [GO:0004725]
HSIVP1_RS09420	- -	cro cl family transcriptional regulator	-2.84	0.00	0.00	0
HSIVP1_RS07790	- -	peptidyl-prolyl cis-trans cyclophilin-type	-4.80	0.00	0.00	peptidyl-prolyl cis-trans isomerase activity [GO:0003755]; protein folding [GO:0006457]
HSIVP1_RS04995	- -	4-oxalocrotonate tautomerase family enzyme	-3.48	0.00	0.00	isomerase activity [GO:0016853]; cellular aromatic compound metabolic process [GO:0006725]
HSIVP1_RS05620	- -	pf06135 family protein	-2.83	0.00	0.00	0
HSIVP1_RS00590	sepF	hypothetical protein	-3.43	0.00	0.00	#N/A
HSIVP1_RS08995	- -	glutamate synthase homotetrameric	-2.86	0.00	0.00	glutamate synthase (NADPH) activity [GO:0004355]; iron-sulfur cluster binding [GO:0051536]
HSIVP1_RS07345	- -	50s ribosomal protein l7 l12	-2.70	0.00	0.00	ribosome [GO:0005840]; structural constituent of ribosome [GO:0003735]; translation [GO:0006412]
HSIVP1_RS07610	Vpar_1550	glutamate dehydrogenase	-5.72	0.00	0.00	oxidoreductase activity, acting on the CH-NH2 group of donors, NAD or NADP as acceptor [GO:0016639]; cellular amino acid metabolic process [GO:0006520]
HSIVP1_RS01330	- -	molybdate abc transporter substrate-binding protein	-4.01	0.00	0.00	metal ion binding [GO:0046872]; molybdate ion transport [GO:0015689]
HSIVP1_RS01560	- -	LktC	-3.31	0.00	0.00	0
HSIVP1_RS04690	- -	aspartyl glutamyl-trna(asn gln) b subunit	-2.98	0.00	0.00	ATP binding [GO:0005524]; glutaminyl-tRNA synthase (glutamine-hydrolyzing) activity [GO:0050567]; transferase activity [GO:0016740]; translation [GO:0006412]
HSIVP1_RS08135	- -	fumarate class ii	-4.65	0.00	0.00	tricarboxylic acid cycle enzyme complex [GO:0045239]; fumarate hydratase activity [GO:0004333]; fumarate metabolic process [GO:0006106]; tricarboxylic acid cycle [GO:0006099]

GeneSymbol (NCBI)	StringDB_locus tags	Gene description	$Log_2FoldChange$[a]	*P* value	P_{adj}[b]	GO terms
HSIVP1_RS03885	gpsA	glycerol-3-phosphate dehydrogenase	2.88	0.00	0.00	glycerol-3-phosphate dehydrogenase complex [GO:0009331]; glycerol-3-phosphate dehydrogenase [NAD(P)+] activity [GO:0047952]; glycerol-3-phosphate dehydrogenase [NAD+] activity [GO:0004367]; NAD binding [GO:0051287]; carbohydrate metabolic process [GO:0005975]; glycerol-3-phosphate biosynthetic process [GO:0046167]; glycerol-3-phosphate catabolic process [GO:0046168]; glycerophospholipid metabolic process [GO:0006650]; phospholipid biosynthetic process [GO:0008654]
HSIVP1_RS03640	Vpar_0790	riboflavin biosynthesis protein	-3.88	0.00	0.00	ATP binding [GO:0005524]; FMN adenylyltransferase activity [GO:0003919]; riboflavin kinase activity [GO:0008531]; FAD biosynthetic process [GO:0006747]; FMN biosynthetic process [GO:0009398]; riboflavin biosynthetic process [GO:0009231]
HSIVP1_RS02820	tmk	thymidylate kinase	-6.21	0.00	0.00	thymidylate kinase activity [GO:0004798]
HSIVP1_RS02130	Vpar_0478	cytochrome d ubiquinol oxidase subunit i	-2.56	0.00	0.00	cytochrome complex [GO:0070069]; integral component of membrane [GO:0016021]; electron transfer activity [GO:0009055]; aerobic electron transport chain [GO:0019646]
HSIVP1_RS00970	Vpar_1293	AraC family transcriptional regulator	-3.57	0.00	0.00	#N/A
HSIVP1_RS09760	Vpar_0979	hemagglutinin	-3.80	0.00	0.00	#N/A
HSIVP1_RS05665	Vpar_1121	thioredoxin	-4.34	0.00	0.00	cell [GO:0005623]; protein disulfide oxidoreductase activity [GO:0015035]; cell redox homeostasis [GO:0045454]; glycerol ether metabolic process [GO:0006662]
HSIVP1_RS02500	- -	abc transporter atp-binding protein	-3.69	0.00	0.00	ATP-binding cassette (ABC) transporter complex [GO:0043190]; ATP binding [GO:0005524]; ATPase activity [GO:0016887]; transmembrane transport [GO:0055085]
HSIVP1_RS04480	- -	pyruvate dehydrogenase	-2.51	0.00	0.00	magnesium ion binding [GO:0000287]; pyruvate dehydrogenase (quinone) activity [GO:0052737]; thiamine pyrophosphate binding [GO:0030976]
HSIVP1_RS09230	Vpar_1772	lactaldehyde reductase	-4.59	0.00	0.00	lactaldehyde reductase activity [GO:0008912]; metal ion binding [GO:0046872]
HSIVP1_RS01565	purE	phosphoribosylaminoimidazole carboxylase	-3.48	0.00	0.00	5-(carboxyamino)imidazole ribonucleotide mutase activity [GO:0034023]; lyase activity [GO:0016829]; 'de novo' IMP biosynthetic process [GO:0006189]
HSIVP1_RS03510	- -	aminopeptidase i zinc metalloprotease	-2.63	0.00	0.00	aminopeptidase activity [GO:0004177]; metallopeptidase activity [GO:0008237]; zinc ion binding [GO:0008270]
HSIVP1_RS01900	murI	glutamate racemase	-3.09	0.00	0.00	glutamate racemase activity [GO:0008881]; cell wall organization [GO:0071555]; peptidoglycan biosynthetic process [GO:0009252]; regulation of cell shape [GO:0008360]
HSIVP1_RS01630	- -	operon protein 2	-5.11	0.00	0.00	0
HSIVP1_RS02535	Vpar_0041	s-layer domain protein	-4.01	0.00	0.00	0
HSIVP1_RS06160	Vpar_1282	threonine synthase	-2.51	0.00	0.00	#N/A
HSIVP1_RS00825	- -	manganese transporter	-2.46	0.00	0.00	metal ion binding [GO:0046872]; cell adhesion [GO:0007155]; metal ion transport [GO:0030001]

GeneSymbol (NCBI)	StringDB_locus tags	Gene description	$Log_2FoldChange^a$	*P* value	P_{adj}^b	GO terms
HSIVP1_RS05450	- -	pf04461 family protein	-4.65	0.00	0.00	0
HSIVP1_RS01250	Vpar_0077	fad dependent oxidoreductase	-2.72	0.00	0.00	0
HSIVP1_RS02475	Vpar_1094	sugar family	2.81	0.00	0.00	arabinose-5-phosphate isomerase activity [GO:0019146]; carbohydrate derivative binding [GO:0097367]; metal ion binding [GO:0046872]; carbohydrate derivative metabolic process [GO:1901135]; carbohydrate metabolic process [GO:0005975]
HSIVP1_RS03075	Vpar_1464	abc transporter	-3.44	0.00	0.00	cytoplasm [GO:0005737]; ATP binding [GO:0005524]; DNA binding [GO:0003677]; valine-tRNA ligase activity [GO:0004832]; valyl-tRNA aminoacylation [GO:0006438]
HSIVP1_RS00965	- -	lysine transporter LysE	-3.44	0.00	0.00	integral component of membrane [GO:0016021]; plasma membrane [GO:0005886]; amino acid transport [GO:0006865]
HSIVP1_RS00630	Vpar_1857	membrane protein	-5.29	0.00	0.00	integral component of membrane [GO:0016021]
HSIVP1_RS05350	- -	conserved domain protein	-2.69	0.00	0.00	0
HSIVP1_RS06350	- -	pf10670 domain protein	-2.48	0.00	0.00	0
HSIVP1_RS01965	- -	hep hag repeat protein	-4.04	0.00	0.00	#N/A
HSIVP1_RS01105	Vpar_1857	membrane protein	-4.53	0.00	0.00	integral component of membrane [GO:0016021]
HSIVP1_RS09535	Vpar_0003	s4 domain protein	-3.77	0.00	0.00	RNA binding [GO:0003723]
HSIVP1_RS09735	- -	conserved domain protein	-4.33	0.00	0.00	0
HSIVP1_RS06495	Vpar_1464	abc transporter	-3.11	0.00	0.00	integral component of membrane [GO:0016021]; plasma membrane [GO:0005886]; transporter activity [GO:0005215]
HSIVP1_RS03460	Vpar_0752	pyruvate carboxylase	-2.33	0.00	0.00	ATP binding [GO:0005524]; biotin binding [GO:0009374]; metal ion binding [GO:0046872]; pyruvate carboxylase activity [GO:0004736]; transferase activity [GO:0016740]; gluconeogenesis [GO:0006094]; pyruvate metabolic process [GO:0006090]
HSIVP1_RS03890	Vpar_0714	rna methyltransferase	2.43	0.00	0.00	methyltransferase activity [GO:0008168]; nucleic acid binding [GO:0003676]; rRNA methylation [GO:0031167]
HSIVP1_RS06745	- -	WYL domain-containing protein	-2.95	0.00	0.00	0
HSIVP1_RS09020	Vpar_1724	phosphatidylglycerophosphatase a	-6.14	0.00	0.00	phosphatidylglycerophosphatase activity [GO:0008962]; lipid metabolic process [GO:0006629]
HSIVP1_RS03445	Vpar_0960	nucleotide-binding protein	-2.31	0.00	0.00	ATP binding [GO:0005524]; ATPase activity [GO:0016887]; iron-sulfur cluster binding [GO:0051536]; metal ion binding [GO:0046872]
HSIVP1_RS02330	- -	fructose-6-phosphate aldolase	-3.25	0.00	0.00	cytoplasm [GO:0005737]; aldehyde-lyase activity [GO:0016832]; sedoheptulose-7-phosphate:D-glyceraldehyde-3-phosphate glyceronetransferase activity [GO:0004801]; carbohydrate metabolic process [GO:0005975]; pentose-phosphate shunt [GO:0006098]
HSIVP1_RS00910	- -	major facilitator family protein	-4.94	0.00	0.00	integral component of membrane [GO:0016021]; transmembrane transport [GO:0055085]
HSIVP1_RS05740	queE	radical sam protein	2.33	0.00	0.00	4 iron, 4 sulfur cluster binding [GO:0051539]; iron ion binding [GO:0005506]; oxidoreductase activity [GO:0016491]

GeneSymbol (NCBI)	StringDB_locus tags	Gene description	$Log_2FoldChange$[a]	*P* value	P_{adj}[b]	GO terms
HSIVP1_RS05660	- -	uptake hydrogenase small subunit	-2.23	0.00	0.00	ferredoxin hydrogenase complex [GO:0009375]; 3 iron, 4 sulfur cluster binding [GO:0051538]; 4 iron, 4 sulfur cluster binding [GO:0051539]; ferredoxin hydrogenase activity [GO:0008901]; hydrogenase (acceptor) activity [GO:0033748]; metal ion binding [GO:0046872]
HSIVP1_RS05835	rplM	ribosomal protein	-2.23	0.00	0.00	ribosome [GO:0005840]
HSIVP1_RS08955	Vpar_0690	dioxygenase	-3.73	0.00	0.00	ferrous iron binding [GO:0008198]; oxidoreductase activity, acting on single donors with incorporation of molecular oxygen [GO:0016701]; zinc ion binding [GO:0008270]; cellular aromatic compound metabolic process [GO:0006725]
HSIVP1_RS03880	- -	acyl-phosphate glycerol 3-phosphate acyltransferase	2.38	0.00	0.00	integral component of membrane [GO:0016021]; plasma membrane [GO:0005886]; acyl-phosphate glycerol-3-phosphate acyltransferase activity [GO:0043772]; phospholipid biosynthetic process [GO:0008654]
HSIVP1_RS09745	- -	hep hag repeat protein	-3.05	0.00	0.00	outer membrane [GO:0019867]; pathogenesis [GO:0009405]
HSIVP1_RS00680	trpB	tryptophan beta subunit	-4.56	0.00	0.00	tryptophan synthase activity [GO:0004834]
HSIVP1_RS05785	Vpar_1212	ppgpp synthetase	2.25	0.00	0.00	GTP diphosphokinase activity [GO:0008728]; kinase activity [GO:0016301]; guanosine tetraphosphate metabolic process [GO:0015969]
HSIVP1_RS01610	- -	2-succinyl-6-hydroxy- -cyclohexadiene-1-carboxylate synthase	-5.21	0.00	0.00	2-succinyl-6-hydroxy-2,4-cyclohexadiene-1-carboxylate synthase activity [GO:0070205]; menaquinone biosynthetic process [GO:0009234]
HSIVP1_RS02125	Vpar_0076	transcriptional regulator	-2.16	0.00	0.00	DNA binding [GO:0003677]
HSIVP1_RS07895	- -	ribonucleoside-diphosphate beta subunit	-3.95	0.00	0.00	metal ion binding [GO:0046872]; ribonucleoside-diphosphate reductase activity, thioredoxin disulfide as acceptor [GO:0004748]; deoxyribonucleotide biosynthetic process [GO:0009263]; DNA replication [GO:0006260]
HSIVP1_RS04540	- -	trna:m u-54 methyltransferase	2.36	0.00	0.00	cytoplasm [GO:0005737]; 5,10-methylenetetrahydrofolate-dependent tRNA (m5U54) methyltransferase activity [GO:0030698]; flavin adenine dinucleotide binding [GO:0050660]; methylenetetrahydrofolate-tRNA-(uracil-5-)-methyltransferase (FADH2-oxidizing) activity [GO:0047151]
HSIVP1_RS09350	- -	sigma- region 4	-5.97	0.00	0.00	DNA-binding transcription factor activity [GO:0003700]; DNA-templated transcription, initiation [GO:0006352]
HSIVP1_RS01945	- -	malate dehydrogenase	-2.20	0.00	0.00	malate dehydrogenase (decarboxylating) (NAD+) activity [GO:0004471]; malate dehydrogenase (decarboxylating) (NADP+) activity [GO:0004473]; metal ion binding [GO:0046872]; NAD binding [GO:0051287]; oxaloacetate decarboxylase activity [GO:0008948]
HSIVP1_RS06715	Vpar_1382	aldehyde dehydrogenase	-2.91	0.00	0.00	lactaldehyde dehydrogenase activity [GO:0008911]
HSIVP1_RS06155	Vpar_1857	membrane protein	-5.54	0.00	0.00	integral component of membrane [GO:0016021]
HSIVP1_RS09465	scpA	chromosome partitioning protein	-3.93	0.00	0.00	DNA binding [GO:0003677]
HSIVP1_RS06905	Vpar_0003	rna-binding protein s4	-2.91	0.00	0.00	RNA binding [GO:0003723]
HSIVP1_RS05430	Vpar_1143	precorrin-4 c -methyltransferase	-3.14	0.00	0.00	precorrin-4 C11-methyltransferase activity [GO:0046026]; cobalamin biosynthetic process [GO:0009236]; oxidation-reduction process [GO:0055114]

GeneSymbol (NCBI)	StringDB_locus tags	Gene description	$Log_2FoldChange$[a]	*P* value	P_{adj}[b]	GO terms
HSIVP1_RS06720	- -	rna-binding transcriptional accessory protein	-3.93	0.00	0.00	nucleic acid binding [GO:0003676]; nucleobase-containing compound metabolic process [GO:0006139]
HSIVP1_RS00335	Vpar_1857	membrane protein	-2.26	0.00	0.00	integral component of membrane [GO:0016021]
HSIVP1_RS07145	rpsK	30s ribosomal protein s11	2.22	0.00	0.00	ribosome [GO:0005840]; rRNA binding [GO:0019843]; structural constituent of ribosome [GO:0003735]; translation [GO:0006412]
HSIVP1_RS04545	topA	dna topoisomerase i	2.23	0.00	0.00	chromosome [GO:0005694]; DNA binding [GO:0003677]; DNA topoisomerase type I activity [GO:0003917]; metal ion binding [GO:0046872]; DNA topological change [GO:0006265]
HSIVP1_RS00190	Vpar_0076	transcriptional regulator	-2.13	0.00	0.00	DNA-binding transcription factor activity [GO:0003700]
HSIVP1_RS03960	Vpar_1359	dna-binding protein	2.25	0.00	0.00	integral component of membrane [GO:0016021]
HSIVP1_RS04580	Vpar_0076	family transcriptional regulator	-2.55	0.00	0.00	DNA binding [GO:0003677]; DNA-binding transcription factor activity [GO:0003700]
HSIVP1_RS01095	Vpar_0303	abc transporter permease	-3.97	0.00	0.00	integral component of membrane [GO:0016021]; transporter activity [GO:0005215]; sodium ion transport [GO:0006814]; transmembrane transport [GO:0055085]
HSIVP1_RS04725	Vpar_0011	rnd transporter	-2.42	0.00	0.00	membrane [GO:0016020]; transporter activity [GO:0005215]; transmembrane transport [GO:0055085]
HSIVP1_RS07105	- -	2-oxoacid ferredoxin oxidoreductase subunit beta	-2.02	0.00	0.00	2-oxoglutarate synthase activity [GO:0047553]; thiamine pyrophosphate binding [GO:0030976]
HSIVP1_RS03260	fabH	3-oxoacyl-	-2.05	0.00	0.00	3-oxo-glutaryl-[acp] methyl ester reductase activity [GO:0102131]; 3-oxo-pimeloyl-[acp] methyl ester reductase activity [GO:0102132]; 3-oxoacyl-[acyl-carrier-protein] reductase (NADPH) activity [GO:0004316]; NAD binding [GO:0051287]; fatty acid biosynthetic process [GO:0006633]
HSIVP1_RS03695	prfB	peptide chain release factor 2	2.13	0.00	0.00	cytoplasm [GO:0005737]; translation release factor activity, codon specific [GO:0016149]
HSIVP1_RS05570	Vpar_1857	membrane protein	-2.17	0.00	0.00	integral component of membrane [GO:0016021]; transporter activity [GO:0005215]; sodium ion transport [GO:0006814]; transmembrane transport [GO:0055085]
HSIVP1_RS06750	Vpar_0835	methyltransferase	-2.95	0.00	0.00	methyltransferase activity [GO:0008168]
HSIVP1_RS08895	- -	pf11148 family protein	-4.83	0.00	0.00	0
HSIVP1_RS00685	- -	tryptophan synthase subunit alpha	-6.04	0.00	0.00	tryptophan synthase activity [GO:0004834]
HSIVP1_RS01410	Vpar_0329	diaminopimelate dehydrogenase	-2.62	0.00	0.00	diaminopimelate dehydrogenase activity [GO:0047850]; nucleotide binding [GO:0000166]; diaminopimelate biosynthetic process [GO:0019877]; lysine biosynthetic process via diaminopimelate [GO:0009089]
HSIVP1_RS05205	- -	sua5 family protein	-4.24	0.00	0.00	cytoplasm [GO:0005737]; ATP binding [GO:0005524]; double-stranded RNA binding [GO:0003725]; L-threonylcarbamoyladenylate synthase [GO:0061710]; tRNA processing [GO:0008033]
HSIVP1_RS08975	Vpar_1716	phosphate acetyltransferase	-2.50	0.00	0.00	phosphate acetyltransferase activity [GO:0008959]
HSIVP1_RS01810	- -	conserved domain protein	-1.99	0.00	0.00	0
HSIVP1_RS04190	miaB	rna family	2.15	0.00	0.00	RNA binding [GO:0003723]; RNA methyltransferase activity [GO:0008173]; RNA processing [GO:0006396]

GeneSymbol (NCBI)	StringDB_locus tags	Gene description	$Log_2FoldChange$[a]	*P* value	P_{adj}[b]	GO terms
HSIVP1_RS06990	- -	twin arginine-targeting protein e family	2.08	0.00	0.00	integral component of plasma membrane [GO:0005887]; TAT protein transport complex [GO:0033281]; protein transmembrane transporter activity [GO:0008320]; protein secretion [GO:0009306]; protein transport by the Tat complex [GO:0043953]
HSIVP1_RS01260	Vpar_0296	aspartate racemase	-3.24	0.00	0.00	racemase activity, acting on amino acids and derivatives [GO:0036361]; cellular amino acid metabolic process [GO:0006520]
HSIVP1_RS02565	pyrB	aspartate carbamoyltransferase	-1.94	0.00	0.00	amino acid binding [GO:0016597]; aspartate carbamoyltransferase activity [GO:0004070]; 'de novo' pyrimidine nucleobase biosynthetic process [GO:0006207]; 'de novo' UMP biosynthetic process [GO:0044205]; cellular amino acid metabolic process [GO:0006520]
HSIVP1_RS05985	Vpar_0994	b12 binding domain protein	-2.14	0.00	0.00	cobalamin binding [GO:0031419]; metal ion binding [GO:0046872]; methylmalonyl-CoA mutase activity [GO:0004494]
HSIVP1_RS09470	- -	sporulation initiation inhibitor protein soj	-2.32	0.00	0.00	0
HSIVP1_RS03425	Vpar_0745	branched chain amino acid aminotransferase	-1.98	0.00	0.00	L-isoleucine transaminase activity [GO:0052656]; L-leucine transaminase activity [GO:0052654]; L-valine transaminase activity [GO:0052655]; branched-chain amino acid metabolic process [GO:0009081]
HSIVP1_RS03100	- -	abc transporter atp-binding protein	-5.59	0.00	0.00	ATP binding [GO:0005524]; ATPase activity [GO:0016887]; branched-chain amino acid transmembrane transporter activity [GO:0015658]
HSIVP1_RS00340	Vpar_1857	membrane protein	-2.03	0.00	0.00	integral component of membrane [GO:0016021]
HSIVP1_RS06050	Vpar_1060	cbs domain protein	-2.00	0.00	0.00	0
HSIVP1_RS04060	- -	trna-2-methylthio-n -dimethylallyladenosine synthase	1.94	0.00	0.00	cytoplasm [GO:0005737]; 4 iron, 4 sulfur cluster binding [GO:0051539]; metal ion binding [GO:0046872]; transferase activity [GO:0016740]; tRNA modification [GO:0006400]
HSIVP1_RS05605	Vpar_1177	aminodeoxychorismate lyase	1.87	0.00	0.00	integral component of plasma membrane [GO:0005887]; lyase activity [GO:0016829]; lytic endotransglycosylase activity [GO:0008932]; cell wall organization [GO:0071555]; peptidoglycan biosynthetic process [GO:0009252]
HSIVP1_RS09145	- -	conserved domain protein	-2.28	0.00	0.00	integral component of membrane [GO:0016021]
HSIVP1_RS06860	sepF	hypothetical protein	-4.82	0.00	0.00	0
HSIVP1_RS01375	- -	selenophosphate synthetase	-4.41	0.00	0.00	ATP binding [GO:0005524]; magnesium ion binding [GO:0000287]; selenide, water dikinase activity [GO:0004756]; selenocysteine biosynthetic process [GO:0016260]
HSIVP1_RS02515	Vpar_1179	resolvase	-3.59	0.00	0.00	0

GeneSymbol (NCBI)	StringDB_locus tags	Gene description	$Log_2FoldChange$[a]	*P* value	P_{adj}[b]	GO terms
HSIVP1_RS02340	- -	udp-n-acetylglucosamine 1-carboxyvinyltransferase	1.94	0.00	0.00	cytoplasm [GO:0005737]; UDP-N-acetylglucosamine 1-carboxyvinyltransferase activity [GO:0008760]; cell cycle [GO:0007049]; cell division [GO:0051301]; cell wall organization [GO:0071555]; peptidoglycan biosynthetic process [GO:0009252]; regulation of cell shape [GO:0008360]; UDP-N-acetylgalactosamine biosynthetic process [GO:0019277]
HSIVP1_RS02060	Vpar_1359	family protein	-2.22	0.00	0.00	cell outer membrane [GO:0009279]; integral component of membrane [GO:0016021]
HSIVP1_RS09765	- -	hep hag repeat protein	-1.90	0.00	0.00	outer membrane [GO:0019867]; pathogenesis [GO:0009405]
HSIVP1_RS04495	polC	dna polymerase iii subunit alpha	1.93	0.00	0.00	cytoplasm [GO:0005737]; 3'-5' exonuclease activity [GO:0008408]; DNA binding [GO:0003677]; DNA-directed DNA polymerase activity [GO:0003887]; DNA-dependent DNA replication [GO:0006261]
HSIVP1_RS09025	- -	succinylarginine dihydrolase	-5.20	0.00	0.00	0
HSIVP1_RS05595	Vpar_1066	peptidase u32	-3.20	0.00	0.00	peptidase activity [GO:0008233]
HSIVP1_RS04370	panD	aspartate 1-decarboxylase	-2.35	0.00	0.00	cytoplasm [GO:0005737]; aspartate 1-decarboxylase activity [GO:0004068]; alanine biosynthetic process [GO:0006523]; pantothenate biosynthetic process [GO:0015940]
HSIVP1_RS02335	- -	fructose- - class ii	-2.50	0.00	0.00	fructose 1,6-bisphosphate 1-phosphatase activity [GO:0042132]; metal ion binding [GO:0046872]; gluconeogenesis [GO:0006094]; glycerol metabolic process [GO:0006071]
HSIVP1_RS03025	- -	nucleoid-associated protein	-2.32	0.00	0.00	nucleoid [GO:0009295]
HSIVP1_RS03250	Vpar_0690	2-nitropropane dioxygenase	-1.85	0.00	0.00	enoyl-[acyl-carrier-protein] reductase (NADH) activity [GO:0004318]; enoyl-[acyl-carrier-protein] reductase activity [GO:0016631]; nitronate monooxygenase activity [GO:0018580]
HSIVP1_RS07475	- -	MULTISPECIES: hypothetical protein	-1.92	0.00	0.00	0
HSIVP1_RS04585	- -	glycine--trna ligase subunit beta	-2.75	0.00	0.01	cytoplasm [GO:0005737]; arginine-tRNA ligase activity [GO:0004814]; ATP binding [GO:0005524]; glycine-tRNA ligase activity [GO:0004820]; arginyl-tRNA aminoacylation [GO:0006420]; glycyl-tRNA aminoacylation [GO:0006426]
HSIVP1_RS05165	tpiA	triose-phosphate isomerase	2.08	0.00	0.01	cytoplasm [GO:0005737]; triose-phosphate isomerase activity [GO:0004807]; gluconeogenesis [GO:0006094]; glycolytic process [GO:0006096]
HSIVP1_RS01890	- -	pf06803 family protein	-1.87	0.00	0.01	0
HSIVP1_RS09245	- -	m50 family	-3.02	0.00	0.01	integral component of membrane [GO:0016021]; metallopeptidase activity [GO:0008237]
HSIVP1_RS00110	Vpar_0075	class i ii	-4.54	0.00	0.01	L-aspartate:2-oxoglutarate aminotransferase activity [GO:0004069]; pyridoxal phosphate binding [GO:0030170]; biosynthetic process [GO:0009058]
HSIVP1_RS06910	proC	pyrroline-5-carboxylate reductase	-2.20	0.00	0.01	cytoplasm [GO:0005737]; pyrroline-5-carboxylate reductase activity [GO:0004735]; L-proline biosynthetic process [GO:0055129]

GeneSymbol (NCBI)	StringDB_locus tags	Gene description	$Log_2FoldChange^a$	*P* value	P_{adj}^b	GO terms
HSIVP1_RS04055	- -	alkaline shock protein asp23 family protein	1.86	0.00	0.01	0
HSIVP1_RS06930	queE	radical sam protein	2.32	0.00	0.01	catalytic activity [GO:0003824]; iron-sulfur cluster binding [GO:0051536]
HSIVP1_RS01100	Vpar_1857	membrane protein	-3.00	0.00	0.01	#N/A
HSIVP1_RS06020	Vpar_0921	polyprenyl synthetase	1.90	0.00	0.01	transferase activity [GO:0016740]; isoprenoid biosynthetic process [GO:0008299]
HSIVP1_RS08225	- -	gdsl-like protein	-2.97	0.00	0.01	0
HSIVP1_RS02810	pth	peptidyl-trna hydrolase	-1.79	0.00	0.01	cytoplasm [GO:0005737]; aminoacyl-tRNA hydrolase activity [GO:0004045]; translation [GO:0006412]
HSIVP1_RS03090	- -	branched-chain amino acid abc permease protein	-3.34	0.00	0.01	integral component of membrane [GO:0016021]; plasma membrane [GO:0005886]; transmembrane transporter activity [GO:0022857]
HSIVP1_RS05290	- -	udp-n-acetylglucosamine--n-acetylmuramyl-pyrophosphoryl-undecaprenol n-acetylglucosamine transferase	1.97	0.00	0.01	plasma membrane [GO:0005886]; UDP-N-acetyl-D-glucosamine:N-acetylmuramoyl-L-alanyl-D-glutamyl-meso-2,6-diaminopimelyl-D-alanyl-D-alanine-diphosphoundecaprenol 4-beta-N-acetylglucosaminlytransferase activity [GO:0051991]; undecaprenyldiphospho-muramoylpentapeptide beta-N-acetylglucosaminyltransferase activity [GO:0050511]; carbohydrate metabolic process [GO:0005975]; cell cycle [GO:0007049]; cell division [GO:0051301]; cell wall organization [GO:0071555]; lipid glycosylation [GO:0030259]; peptidoglycan biosynthetic process [GO:0009252]; regulation of cell shape [GO:0008360]
HSIVP1_RS03520	Vpar_0309	abc transporter substrate binding protein	-2.03	0.00	0.01	0
HSIVP1_RS03405	Vpar_0740	antibiotic biosynthesis monooxygenase	-1.98	0.00	0.01	0
HSIVP1_RS08795	Vpar_1681	replicative dna helicase	-2.57	0.00	0.01	primosome complex [GO:1990077]; ATP binding [GO:0005524]; DNA binding [GO:0003677]; DNA helicase activity [GO:0003678]; DNA replication, synthesis of RNA primer [GO:0006269]
HSIVP1_RS09585	Vpar_0835	methyltransferase	-2.52	0.00	0.01	0
HSIVP1_RS07025	- -	major facilitator family protein	-1.71	0.00	0.01	#N/A
HSIVP1_RS08110	- -	cysteine-rich domain protein	-2.91	0.00	0.01	0
HSIVP1_RS09415	sepF	hypothetical protein	-1.72	0.00	0.01	0
HSIVP1_RS05200	- -	low molecular weight phosphotyrosine protein phosphatase	-2.65	0.00	0.01	protein tyrosine phosphatase activity [GO:0004725]
HSIVP1_RS06060	- -	metallo-beta-lactamase domain protein	2.23	0.00	0.01	hydrolase activity [GO:0016787]
HSIVP1_RS00440	Vpar_1464	abc transporter	-2.71	0.00	0.01	ATP binding [GO:0005524]; ATPase activity [GO:0016887]
HSIVP1_RS09390	- -	hypothetical protein HSIVP1_1243	-2.23	0.00	0.01	#N/A
HSIVP1_RS07930	Vpar_1614	chorismate mutase	-1.88	0.00	0.01	cytoplasm [GO:0005737]; chorismate mutase activity [GO:0004106]; prephenate dehydratase activity [GO:0004664]; chorismate metabolic process [GO:0046417]; L-phenylalanine biosynthetic process [GO:0009094]
HSIVP1_RS00505	Vpar_0134	carbon starvation protein	-1.64	0.00	0.01	integral component of membrane [GO:0016021]; cellular response to starvation [GO:0009267]
HSIVP1_RS02240	ftsZ	cell division protein	1.79	0.00	0.01	0

GeneSymbol (NCBI)	StringDB_locus tags	Gene description	$Log_2FoldChange$[a]	*P* value	P_{adj}[b]	GO terms
HSIVP1_RS09570	- -	rnd hae1 hme permease protein	-1.76	0.00	0.01	#N/A
HSIVP1_RS00930	Vpar_0224	nitrate reductase molybdenum cofactor assembly chaperone	-1.67	0.00	0.01	nitrate reductase activity [GO:0008940]; unfolded protein binding [GO:0051082]; chaperone-mediated protein complex assembly [GO:0051131]
HSIVP1_RS03965	rsmG	16s rrna methyltransferase	1.75	0.00	0.01	cytoplasm [GO:0005737]; RNA binding [GO:0003723]; rRNA methyltransferase activity [GO:0008649]; regulation of transcription, DNA-templated [GO:0006355]
HSIVP1_RS04930	- -	sporulation and spore germination	1.73	0.00	0.01	0
HSIVP1_RS05160	- -	phosphoglycerate mutase(2,3-diphosphoglycerate-independent)	1.72	0.00	0.01	cytoplasm [GO:0005737]; 2,3-bisphosphoglycerate-independent phosphoglycerate mutase activity [GO:0046537]; manganese ion binding [GO:0030145]; glucose catabolic process [GO:0006007]; glycolytic process [GO:0006096]
HSIVP1_RS08370	Vpar_0002	dna polymerase iii	2.36	0.00	0.01	0
HSIVP1_RS04390	Vpar_1019	domain protein	-3.22	0.00	0.01	0
HSIVP1_RS01935	- -	phosphodiesterase family protein	-2.97	0.00	0.02	hydrolase activity [GO:0016787]; metal ion binding [GO:0046872]
HSIVP1_RS01830	- -	mfs transporter	-1.97	0.00	0.02	integral component of membrane [GO:0016021]; transmembrane transport [GO:0055085]
HSIVP1_RS01455	- -	conserved domain protein	1.64	0.00	0.02	0
HSIVP1_RS07470	- -	cyclic nucleotide-binding domain protein	-1.74	0.00	0.02	DNA binding [GO:0003677]; regulation of transcription, DNA-templated [GO:0006355]; transcription, DNA-templated [GO:0006351]
HSIVP1_RS00690	- -	wd repeat-containing protein 60	-3.35	0.00	0.02	0
HSIVP1_RS05765	hisS	histidine--trna ligase	-2.04	0.00	0.02	cytoplasm [GO:0005737]; ATP binding [GO:0005524]; histidine-tRNA ligase activity [GO:0004821]; histidyl-tRNA aminoacylation [GO:0006427]
HSIVP1_RS05915	- -	conserved domain protein	-2.11	0.00	0.02	#N/A
HSIVP1_RS00455	- -	histidinol-phosphatase	-3.42	0.00	0.02	#N/A
HSIVP1_RS01570	- -	phosphoribosylaminoimidazolesuccinocarboxamide synthase	-2.67	0.00	0.02	ATP binding [GO:0005524]; phosphoribosylaminoimidazolesuccinocarboxamide synthase activity [GO:0004639]; 'de novo' IMP biosynthetic process [GO:0006189]; cobalamin biosynthetic process [GO:0009236]
HSIVP1_RS03955	fmt	methionyl-trna formyltransferase	2.15	0.00	0.02	methionyl-tRNA formyltransferase activity [GO:0004479]
HSIVP1_RS02600	- -	MULTISPECIES: hypothetical protein	1.59	0.00	0.02	0
HSIVP1_RS09580	- -	membrane pf01595 family	-1.81	0.00	0.02	integral component of membrane [GO:0016021]; flavin adenine dinucleotide binding [GO:0050660]
HSIVP1_RS05210	- -	protein-(glutamine-n5) release factor-specific	1.75	0.00	0.02	nucleic acid binding [GO:0003676]; protein-(glutamine-N5) methyltransferase activity [GO:0102559]; protein-glutamine N-methyltransferase activity [GO:0036009]; peptidyl-glutamine methylation [GO:0018364]
HSIVP1_RS01325	Vpar_0308	auxin efflux carrier family protein	-2.12	0.00	0.02	integral component of membrane [GO:0016021]; transmembrane transport [GO:0055085]
HSIVP1_RS04830	Vpar_1019	domain protein	1.95	0.00	0.02	0
HSIVP1_RS05790	Vpar_1213	single-stranded-dna-specific exonuclease	1.64	0.00	0.02	5'-3' exonuclease activity [GO:0008409]; nucleic acid binding [GO:0003676]; DNA recombination [GO:0006310]; DNA repair [GO:0006281]

GeneSymbol (NCBI)	StringDB_locus tags	Gene description	$Log_2FoldChange$[a]	*P* value	P_{adj}[b]	GO terms
HSIVP1_RS06245	guaC	gmp reductase	-2.23	0.00	0.02	cytoplasm [GO:0005737]; GMP reductase complex [GO:1902560]; [isocitrate dehydrogenase (NADP+)] kinase activity [GO:0008772]; GMP reductase activity [GO:0003920]; phosphatase activity [GO:0016791]; glucose metabolic process [GO:0006006]; purine nucleotide metabolic process [GO:0006163]
HSIVP1_RS08915	Vpar_1704	succinate dehydrogenase and fumarate reductase iron-sulfur protein	-3.50	0.00	0.02	2 iron, 2 sulfur cluster binding [GO:0051537]; electron transfer activity [GO:0009055]; succinate dehydrogenase activity [GO:0000104]; tricarboxylic acid cycle [GO:0006099]
HSIVP1_RS06880	Vpar_1423	delta-aminolevulinic acid dehydratase	-1.91	0.00	0.02	metal ion binding [GO:0046872]; porphobilinogen synthase activity [GO:0004655]; porphyrin-containing compound biosynthetic process [GO:0006779]
HSIVP1_RS06240	Vpar_1300	proton-coupled thiamine transporter	-2.50	0.00	0.02	integral component of membrane [GO:0016021]; plasma membrane [GO:0005886]; thiamine transmembrane transporter activity [GO:0015234]; thiamine transport [GO:0015888]
HSIVP1_RS01550	xpt	xanthine phosphoribosyltransferase	-1.74	0.00	0.02	cytoplasm [GO:0005737]; xanthine phosphoribosyltransferase activity [GO:0000310]; purine ribonucleoside salvage [GO:0006166]; xanthine metabolic process [GO:0046110]; XMP salvage [GO:0032265]
HSIVP1_RS05875	Vpar_1224	penicillin-binding protein 2	2.30	0.00	0.02	integral component of membrane [GO:0016021]; penicillin binding [GO:0008658]; peptidoglycan glycosyltransferase activity [GO:0008955]; serine-type D-Ala-D-Ala carboxypeptidase activity [GO:0009002]; cell division [GO:0051301]; peptidoglycan biosynthetic process [GO:0009252]
HSIVP1_RS05770	- -	coproporphyrinogen dehydrogenase	-1.58	0.00	0.02	catalytic activity [GO:0003824]; iron-sulfur cluster binding [GO:0051536]
HSIVP1_RS04315	xseA	exodeoxyribonuclease small subunit	-2.96	0.00	0.03	cytoplasm [GO:0005737]; exodeoxyribonuclease VII complex [GO:0009318]; exodeoxyribonuclease VII activity [GO:0008855]; DNA catabolic process [GO:0006308]
HSIVP1_RS07850	secY	hydrophobic domain protein	-3.87	0.00	0.03	integral component of membrane [GO:0016021]
HSIVP1_RS04550	Vpar_0969	dna protecting protein	-2.73	0.00	0.03	#N/A
HSIVP1_RS02310	- -	guanine permease	2.04	0.00	0.03	integral component of membrane [GO:0016021]; transmembrane transporter activity [GO:0022857]
HSIVP1_RS02585	- -	dihydropicolinate synthase	1.65	0.00	0.03	2 iron, 2 sulfur cluster binding [GO:0051537]; electron transfer activity [GO:0009055]; flavin adenine dinucleotide binding [GO:0050660]; metal ion binding [GO:0046872]; 'de novo' UMP biosynthetic process [GO:0044205]
HSIVP1_RS08120	Vpar_0041	s-layer protein	-1.71	0.01	0.03	0
HSIVP1_RS01895	- -	acyl- thioester family	1.85	0.01	0.03	hydrolase activity [GO:0016787]
HSIVP1_RS08165	- -	galactose-1-phosphate uridylyltransferase	-2.40	0.01	0.03	UDP-glucose:hexose-1-phosphate uridylyltransferase activity [GO:0008108]; UTP:galactose-1-phosphate uridylyltransferase activity [GO:0017103]; zinc ion binding [GO:0008270]; galactose catabolic process via UDP-galactose [GO:0033499]

GeneSymbol (NCBI)	StringDB_locus tags	Gene description	$Log_2FoldChange$[a]	*P* value	P_{adj}[b]	GO terms
HSIVP1_RS06725	Vpar_1384	dihydrofolate reductase	-3.16	0.01	0.03	dihydrofolate reductase activity [GO:0004146]; NADP binding [GO:0050661]; glycine biosynthetic process [GO:0006545]; one-carbon metabolic process [GO:0006730]; tetrahydrofolate biosynthetic process [GO:0046654]
HSIVP1_RS02625	Vpar_1359	dna-binding protein	1.71	0.01	0.03	0
HSIVP1_RS02255	tilS	trna -lysidine synthetase	2.10	0.01	0.03	cytoplasm [GO:0005737]; ATP binding [GO:0005524]; ligase activity, forming carbon-nitrogen bonds [GO:0016879]; tRNA modification [GO:0006400]
HSIVP1_RS03050	Vpar_0658	glucose-6-phosphate isomerase	1.57	0.01	0.03	#N/A
HSIVP1_RS02470	- -	3-deoxy-8-phosphooctulonate synthase	1.50	0.01	0.03	cytoplasm [GO:0005737]; 3-deoxy-8-phosphooctulonate synthase activity [GO:0008676]; keto-3-deoxy-D-manno-octulosonic acid biosynthetic process [GO:0019294]
HSIVP1_RS04760	secY	preprotein subunit	1.65	0.01	0.03	integral component of membrane [GO:0016021]; P-P-bond-hydrolysis-driven protein transmembrane transporter activity [GO:0015450]; protein secretion [GO:0009306]
HSIVP1_RS08055	Vpar_0011	efflux rnd mfp subunit	1.50	0.01	0.03	membrane [GO:0016020]; transporter activity [GO:0005215]; transmembrane transport [GO:0055085]
HSIVP1_RS03730	ftsZ	cell division protein	1.55	0.01	0.03	integral component of membrane [GO:0016021]; plasma membrane [GO:0005886]; cell cycle [GO:0007049]; cell division [GO:0051301]
HSIVP1_RS02790	Vpar_0605	3d domain protein	1.51	0.01	0.03	outer membrane [GO:0019867]; hydrolase activity, hydrolyzing O-glycosyl compounds [GO:0004553]; peptidoglycan turnover [GO:0009254]
HSIVP1_RS08205	- -	lipid rv2252 family	-1.48	0.01	0.03	integral component of membrane [GO:0016021]; NAD+ kinase activity [GO:0003951]
HSIVP1_RS08875	Vpar_0074	TonB-dependent receptor	-1.55	0.01	0.04	cell outer membrane [GO:0009279]; integral component of membrane [GO:0016021]
HSIVP1_RS05320	- -	16s rrna (cytosine -n)-methyltransferase	1.60	0.01	0.04	cytoplasm [GO:0005737]; rRNA (cytosine-N4-)-methyltransferase activity [GO:0071424]; rRNA base methylation [GO:0070475]
HSIVP1_RS07905	- -	gnat family acetyltransferase	-1.73	0.01	0.04	glucosamine 6-phosphate N-acetyltransferase activity [GO:0004343]; UDP-N-acetylglucosamine biosynthetic process [GO:0006048]
HSIVP1_RS05540	sepF	hypothetical protein	-2.92	0.01	0.04	#N/A
HSIVP1_RS01805	gatB	glutamyl-trna amidotransferase subunit a	-1.43	0.01	0.04	0
HSIVP1_RS06775	- -	mfs transporter	-2.90	0.01	0.04	integral component of membrane [GO:0016021]; transmembrane transport [GO:0055085]
HSIVP1_RS08280	sepF	hypothetical protein	-3.35	0.01	0.04	#N/A
HSIVP1_RS05000	recD2	atp-dependent dna helicase	-4.10	0.01	0.04	ATP binding [GO:0005524]; ATP-dependent DNA helicase activity [GO:0004003]; nucleic acid binding [GO:0003676]; DNA recombination [GO:0006310]; DNA repair [GO:0006281]
HSIVP1_RS02275	- -	bifunctional biotin--	1.66	0.01	0.04	ATP binding [GO:0005524]; biotin-[acetyl-CoA-carboxylase] ligase activity [GO:0004077]; DNA binding [GO:0003677]; protein biotinylation [GO:0009305]; regulation of transcription, DNA-templated [GO:0006355]

GeneSymbol (NCBI)	StringDB_locus tags	Gene description	$Log_2FoldChange$[a]	*P* value	P_{adj}[b]	GO terms
HSIVP1_RS02250	- -	ppx phosphatase family protein	1.97	0.01	0.04	exopolyphosphatase activity [GO:0004309]
HSIVP1_RS05520	Vpar_1000	dna helicase	1.48	0.01	0.04	#N/A
HSIVP1_RS05910	ackA	acetate kinase	-1.43	0.01	0.04	cytoplasm [GO:0005737]; acetate kinase activity [GO:0008776]; ATP binding [GO:0005524]; magnesium ion binding [GO:0000287]; acetyl-CoA biosynthetic process [GO:0006085]; organic acid metabolic process [GO:0006082]
HSIVP1_RS01910	- -	f420-0:gamma-glutamyl ligase	-1.74	0.01	0.04	0
HSIVP1_RS03485	- -	adp-glyceromanno-heptose 6-epimerase	-1.70	0.01	0.04	ADP-glyceromanno-heptose 6-epimerase activity [GO:0008712]; NADP binding [GO:0050661]; ADP-L-glycero-beta-D-manno-heptose biosynthetic process [GO:0097171]; carbohydrate metabolic process [GO:0005975]
HSIVP1_RS05990	Vpar_1248	methylmalonyl- mutase	-3.09	0.01	0.04	cobalamin binding [GO:0031419]; methylmalonyl-CoA mutase activity [GO:0004494]
HSIVP1_RS00105	Vpar_0074	TonB-dependent receptor	-2.35	0.01	0.05	cell outer membrane [GO:0009279]; integral component of membrane [GO:0016021]
HSIVP1_RS01010	Vpar_0309	abc transporter substrate-binding protein	-1.73	0.01	0.05	metal ion binding [GO:0046872]; cell adhesion [GO:0007155]; metal ion transport [GO:0030001]
HSIVP1_RS06250	- -	major facilitator family protein	-2.14	0.01	0.05	integral component of membrane [GO:0016021]; transmembrane transport [GO:0055085]
HSIVP1_RS02365	- -	peptidase s55	1.42	0.01	0.05	0
HSIVP1_RS05330	Vpar_1121	thioredoxin	-1.44	0.01	0.05	cell [GO:0005623]; protein disulfide oxidoreductase activity [GO:0015035]; cell redox homeostasis [GO:0045454]; glycerol ether metabolic process [GO:0006662]
HSIVP1_RS03530	Vpar_0303	abc transporter permease	-2.85	0.01	0.05	integral component of membrane [GO:0016021]; plasma membrane [GO:0005886]; transmembrane transporter activity [GO:0022857]
HSIVP1_RS08185	- -	molybdopterin-binding protein	-1.64	0.01	0.05	0
HSIVP1_RS01080	Vpar_0254	formate acetyltransferase	-1.36	0.01	0.05	cytoplasm [GO:0005737]; formate C-acetyltransferase activity [GO:0008861]; lyase activity [GO:0016829]; carbohydrate metabolic process [GO:0005975]
HSIVP1_RS05395	Vpar_0309	abc transporter substrate-binding protein	-1.46	0.01	0.05	0
HSIVP1_RS06830	- -	pf01863 family protein	-4.14	0.01	0.05	0
HSIVP1_RS01855	Vpar_0745	amino acid aminotransferase	-1.59	0.01	0.05	D-alanine:2-oxoglutarate aminotransferase activity [GO:0047810]
HSIVP1_RS04745	Vpar_1072	family protein	-2.24	0.01	0.05	integral component of membrane [GO:0016021]
HSIVP1_RS07515	- -	atp synthase epsilon subunit	-1.40	0.01	0.05	plasma membrane [GO:0005886]; proton-transporting ATP synthase complex, catalytic core F(1) [GO:0045261]; ATP binding [GO:0005524]; proton-transporting ATP synthase activity, rotational mechanism [GO:0046933]; ATP synthesis coupled proton transport [GO:0015986]
HSIVP1_RS09565	- -	heavy-metal-binding domain protein	-2.25	0.01	0.05	0
HSIVP1_RS01275	glmS	glutamine--fructose-6-phosphate aminotransferase	-1.34	0.01	0.05	#N/A
HSIVP1_RS02985	- -	MULTISPECIES: hypothetical protein	-1.36	0.01	0.05	0

GeneSymbol (NCBI)	StringDB_locus tags	Gene description	$Log_2FoldChange^a$	*P* value	P_{adj}^{b}	GO terms
HSIVP1_RS04115	hrcA	heat-inducible transcription repressor	1.90	0.01	0.05	DNA binding [GO:0003677]; negative regulation of transcription, DNA-templated [GO:0045892]
HSIVP1_RS03700	- -	mg032 mg096 mg288 family 1	1.37	0.01	0.06	0
HSIVP1_RS06985	- -	heavy metal-associated domain protein	1.59	0.01	0.06	metal ion binding [GO:0046872]; metal ion transport [GO:0030001]
HSIVP1_RS04910	Vpar_1039	ribosomal-protein-alanine acetyltransferase	1.45	0.01	0.06	cytoplasm [GO:0005737]; acetyltransferase activity [GO:0016407]; N-terminal protein amino acid acetylation [GO:0006474]
HSIVP1_RS01705	- -	oxaloacetate decarboxylase	-1.35	0.01	0.06	pyruvate carboxylase activity [GO:0004736]; transferase activity [GO:0016740]
HSIVP1_RS01860	Vpar_0258	methionine abc transporter atp-binding protein	-1.55	0.01	0.06	plasma membrane [GO:0005886]; amino acid-transporting ATPase activity [GO:0015424]; ATP binding [GO:0005524]; methionine transport [GO:0015821]
HSIVP1_RS05295	murD	udp-n-acetylmuramoylalanine--d-glutamate ligase	1.53	0.01	0.06	cytoplasm [GO:0005737]; ATP binding [GO:0005524]; tetrahydrofolylpolyglutamate synthase activity [GO:0004326]; UDP-N-acetylmuramoylalanine-D-glutamate ligase activity [GO:0008764]; cell cycle [GO:0007049]; cell division [GO:0051301]; cell wall organization [GO:0071555]; peptidoglycan biosynthetic process [GO:0009252]; regulation of cell shape [GO:0008360]
HSIVP1_RS02735	Vpar_0597	5-formyltetrahydrofolate cyclo-ligase	1.73	0.01	0.06	5-formyltetrahydrofolate cyclo-ligase activity [GO:0030272]; ATP binding [GO:0005524]; metal ion binding [GO:0046872]
HSIVP1_RS02575	carA	carbamoyl-phosphate small subunit	1.43	0.01	0.06	ATP binding [GO:0005524]; carbamoyl-phosphate synthase (glutamine-hydrolyzing) activity [GO:0004088]; 'de novo' pyrimidine nucleobase biosynthetic process [GO:0006207]; 'de novo' UMP biosynthetic process [GO:0044205]; arginine biosynthetic process [GO:0006526]; glutamine metabolic process [GO:0006541]
HSIVP1_RS04825	Vpar_1022	atp--guanido phosphotransferase	2.20	0.01	0.06	ATP binding [GO:0005524]; kinase activity [GO:0016301]; phosphotransferase activity, nitrogenous group as acceptor [GO:0016775]
HSIVP1_RS05170	pgk	phosphoglycerate kinase	1.41	0.01	0.06	cytoplasm [GO:0005737]; ATP binding [GO:0005524]; phosphoglycerate kinase activity [GO:0004618]; glycolytic process [GO:0006096]
HSIVP1_RS02315	Vpar_0512	guanine deaminase	2.56	0.01	0.06	guanine deaminase activity [GO:0008892]; zinc ion binding [GO:0008270]; guanine catabolic process [GO:0006147]
HSIVP1_RS09105	Vpar_1745	catalase	-2.72	0.01	0.06	catalase activity [GO:0004096]; heme binding [GO:0020037]; metal ion binding [GO:0046872]; hydrogen peroxide catabolic process [GO:0042744]; response to oxidative stress [GO:0006979]
HSIVP1_RS04840	speD	s-adenosylmethionine decarboxylase	-3.70	0.01	0.06	adenosylmethionine decarboxylase activity [GO:0004014]; S-adenosylmethioninamine biosynthetic process [GO:0006557]; spermidine biosynthetic process [GO:0008295]

GeneSymbol (NCBI)	StringDB_locus tags	Gene description	$Log_2FoldChange$[a]	*P* value	P_{adj}[b]	GO terms
HSIVP1_RS02455	- -	3-deoxy-d-manno-octulosonic-acid transferase	1.45	0.01	0.06	integral component of membrane [GO:0016021]; transferase activity [GO:0016740]
HSIVP1_RS02545	- -	glmz -inactivating ntpase	-1.72	0.01	0.06	ATP binding [GO:0005524]; GTP binding [GO:0005525]; kinase activity [GO:0016301]
HSIVP1_RS09620	- -	glycosyl transferases group 1 family protein	1.67	0.01	0.06	transferase activity [GO:0016740]
HSIVP1_RS03905	Vpar_0838	fibronectin-binding protein a domain protein	1.40	0.01	0.06	#N/A
HSIVP1_RS09030	Vpar_1726	alkylphosphonate utilization operon protein	-1.46	0.01	0.06	0
HSIVP1_RS06485	- -	pts maltose transporter subunit iibc	-1.59	0.01	0.06	protein-N(PI)-phosphohistidine-sugar phosphotransferase activity [GO:0008982]; phosphoenolpyruvate-dependent sugar phosphotransferase system [GO:0009401]
HSIVP1_RS00570	Vpar_1072	membrane protein	-1.74	0.01	0.06	integral component of membrane [GO:0016021]
HSIVP1_RS06015	Vpar_1010	preprotein translocase subunit	1.42	0.01	0.06	integral component of plasma membrane [GO:0005887]; TAT protein transport complex [GO:0033281]; protein transmembrane transporter activity [GO:0008320]; protein transport by the Tat complex [GO:0043953]
HSIVP1_RS09155	Vpar_0058	proton channel family protein	1.55	0.01	0.06	integral component of membrane [GO:0016021]; protein transport [GO:0015031]
HSIVP1_RS03735	Vpar_0809	peptidase m23	1.42	0.01	0.06	0
HSIVP1_RS01190	surE	5 -nucleotidase	-1.70	0.01	0.06	0
HSIVP1_RS02870	Vpar_0605	3d domain protein	1.82	0.01	0.06	integral component of membrane [GO:0016021]; outer membrane [GO:0019867]; hydrolase activity, hydrolyzing O-glycosyl compounds [GO:0004553]; peptidoglycan turnover [GO:0009254]
HSIVP1_RS05215	prfA	peptide chain release factor 1	1.36	0.01	0.06	cytoplasm [GO:0005737]; translation release factor activity, codon specific [GO:0016149]
HSIVP1_RS02505	Vpar_0273	permease	-1.95	0.01	0.06	integral component of membrane [GO:0016021]
HSIVP1_RS02400	- -	gram-positive signal peptide ysirk family	-1.65	0.01	0.07	unfolded protein binding [GO:0051082]
HSIVP1_RS03855	Vpar_0828	fad-dependent oxidoreductase	1.41	0.01	0.07	0
HSIVP1_RS06225	Vpar_1297	cupin domain protein	-2.23	0.02	0.07	0
HSIVP1_RS06500	- -	iron(III) dicitrate transport ATP-binding protein FecE	1.65	0.02	0.07	ATP binding [GO:0005524]; ATPase activity [GO:0016887]
HSIVP1_RS05690	Vpar_1203	radical sam protein	-2.94	0.02	0.07	4 iron, 4 sulfur cluster binding [GO:0051539]; carbon-nitrogen lyase activity [GO:0016840]; magnesium ion binding [GO:0000287]; S-adenosyl-L-methionine binding [GO:1904047]; queuosine biosynthetic process [GO:0008616]
HSIVP1_RS03085	Vpar_0665	branched-chain amino acid abc permease protein	-1.52	0.02	0.07	integral component of membrane [GO:0016021]; plasma membrane [GO:0005886]; transmembrane transporter activity [GO:0022857]
HSIVP1_RS00205	- -	electron transporter	-2.01	0.02	0.07	electron transfer activity [GO:0009055]
HSIVP1_RS05390	Vpar_1132	cobalt chelatase	-1.41	0.02	0.07	metal ion binding [GO:0046872]; sirohydrochlorin cobaltochelatase activity [GO:0016852]; anaerobic cobalamin biosynthetic process [GO:0019251]
HSIVP1_RS00520	Vpar_0136	copper amine oxidase	-1.88	0.02	0.07	0
HSIVP1_RS08160	mutL	dna mismatch repair protein	1.59	0.02	0.07	8-oxo-7,8-dihydroguanosine triphosphate pyrophosphatase activity [GO:0008413]; DNA repair [GO:0006281]

GeneSymbol (NCBI)	StringDB_locus tags	Gene description	$Log_2FoldChange^a$	*P* value	P_{adj}^b	GO terms
HSIVP1_RS02030	Vpar_1072	membrane protein	-1.68	0.02	0.07	integral component of membrane [GO:0016021]; plasma membrane [GO:0005886]; metal ion transmembrane transporter activity [GO:0046873]
HSIVP1_RS04510	Vpar_0961	competence protein	1.68	0.02	0.07	integral component of membrane [GO:0016021]; DNA binding [GO:0003677]; DNA repair [GO:0006281]
HSIVP1_RS05005	rpmB	50s ribosomal protein l28	1.38	0.02	0.07	ribosome [GO:0005840]; structural constituent of ribosome [GO:0003735]; translation [GO:0006412]
HSIVP1_RS02375	Vpar_0807	abc atp-binding protein	1.43	0.02	0.07	ATP binding [GO:0005524]; ATPase activity [GO:0016887]
HSIVP1_RS05400	- -	ferrichrome abc atp-binding protein	-1.36	0.02	0.07	ATP binding [GO:0005524]; ATPase activity [GO:0016887]
HSIVP1_RS00755	guaA	gmp synthetase	-1.52	0.02	0.08	ATP binding [GO:0005524]; GMP synthase (glutamine-hydrolyzing) activity [GO:0003922]; pyrophosphatase activity [GO:0016462]; glutamine metabolic process [GO:0006541]; GMP biosynthetic process [GO:0006177]
HSIVP1_RS03465	Vpar_0398	family transcriptional regulator	1.30	0.02	0.08	ATP binding [GO:0005524]; DNA binding [GO:0003677]; zinc ion binding [GO:0008270]; negative regulation of transcription, DNA-templated [GO:0045892]
HSIVP1_RS08255	Vpar_0006	hypothetical protein	1.27	0.02	0.08	0
HSIVP1_RS09015	Vpar_0112	isochorismatase family protein	-1.96	0.02	0.08	nicotinamidase activity [GO:0008936]
HSIVP1_RS03455	- -	aminopeptidase i zinc metalloprotease	-1.79	0.02	0.08	aminopeptidase activity [GO:0004177]; metallopeptidase activity [GO:0008237]; zinc ion binding [GO:0008270]
HSIVP1_RS09400	Vpar_0993	conserved domain protein	1.54	0.02	0.08	integral component of membrane [GO:0016021]
HSIVP1_RS07605	Vpar_1549	amino acid carrier protein	-1.32	0.02	0.08	integral component of membrane [GO:0016021]; plasma membrane [GO:0005886]; alanine:sodium symporter activity [GO:0015655]
HSIVP1_RS04145	- -	trna (n -l-threonylcarbamoyladenosine -c)-methylthiotransferase	1.28	0.02	0.08	4 iron, 4 sulfur cluster binding [GO:0051539]; N6-threonylcarbomyladenosine methylthiotransferase activity [GO:0035598]
HSIVP1_RS09135	Vpar_1753	adenylosuccinate lyase	1.39	0.02	0.08	(S)-2-(5-amino-1-(5-phospho-D-ribosyl)imidazole-4-carboxamido)succinate AMP-lyase (fumarate-forming) activity [GO:0070626]; N6-(1,2-dicarboxyethyl)AMP AMP-lyase (fumarate-forming) activity [GO:0004018]; 'de novo' AMP biosynthetic process [GO:0044208]; 'de novo' IMP biosynthetic process [GO:0006189]
HSIVP1_RS06535	- -	galactosyldiacylglycerol synthase	1.32	0.02	0.08	#N/A
HSIVP1_RS05945	Vpar_1290	dass family	-1.25	0.02	0.08	integral component of membrane [GO:0016021]; transporter activity [GO:0005215]; sodium ion transport [GO:0006814]; transmembrane transport [GO:0055085]
HSIVP1_RS09595	- -	arylsulfatase	-2.08	0.02	0.08	integral component of membrane [GO:0016021]; sulfuric ester hydrolase activity [GO:0008484]
HSIVP1_RS03950	def	peptide deformylase	1.43	0.02	0.08	#N/A
HSIVP1_RS01130	- -	peptidase propeptide and ypeb domain protein	-1.22	0.02	0.08	0
HSIVP1_RS05695	- -	queuosine biosynthesis protein	-2.04	0.02	0.09	lyase activity [GO:0016829]; metal ion binding [GO:0046872]; queuosine biosynthetic process [GO:0008616]
HSIVP1_RS04565	rnhB	ribonuclease hii	1.45	0.02	0.09	cytoplasm [GO:0005737]; manganese ion binding [GO:0030145]; RNA binding [GO:0003723]; RNA-DNA hybrid ribonuclease activity [GO:0004523]; RNA catabolic process [GO:0006401]

GeneSymbol (NCBI)	StringDB_locus tags	Gene description	$Log_2FoldChange$[a]	*P* value	P_{adj}[b]	GO terms
HSIVP1_RS01460	trmB	trna (guanine-n -)-methyltransferase	1.22	0.02	0.09	tRNA (guanine-N7-)-methyltransferase activity [GO:0008176]
HSIVP1_RS03440	Vpar_0267	class i ii	-3.75	0.02	0.10	L-aspartate:2-oxoglutarate aminotransferase activity [GO:0004069]; pyridoxal phosphate binding [GO:0030170]; biosynthetic process [GO:0009058]
HSIVP1_RS03705	Vpar_1072	membrane protein	1.25	0.02	0.10	integral component of membrane [GO:0016021]
HSIVP1_RS01305	Vpar_0006	hypothetical protein	-1.32	0.02	0.10	#N/A
HSIVP1_RS02700	ruvC	crossover junction endodeoxyribonuclease	1.80	0.02	0.10	crossover junction endodeoxyribonuclease activity [GO:0008821]; magnesium ion binding [GO:0000287]; nucleic acid binding [GO:0003676]; DNA recombination [GO:0006310]; DNA repair [GO:0006281]
HSIVP1_RS00875	bioB	biotin synthase	-1.84	0.02	0.10	2 iron, 2 sulfur cluster binding [GO:0051537]; 4 iron, 4 sulfur cluster binding [GO:0051539]; biotin synthase activity [GO:0004076]; iron ion binding [GO:0005506]; biotin biosynthetic process [GO:0009102]
HSIVP1_RS07755	Vpar_0476	rrf2 family transcriptional regulator	-1.52	0.02	0.10	DNA binding [GO:0003677]
HSIVP1_RS02465	kdsB	3-deoxy-manno-octulosonate cytidylyltransferase	1.19	0.03	0.10	cytoplasm [GO:0005737]; 3-deoxy-manno-octulosonate cytidylyltransferase activity [GO:0008690]; CMP-keto-3-deoxy-D-manno-octulosonic acid biosynthetic process [GO:0033468]; lipopolysaccharide biosynthetic process [GO:0009103]
HSIVP1_RS02445	Vpar_0538	lipid-a-disaccharide synthase	1.24	0.03	0.10	lipid-A-disaccharide synthase activity [GO:0008915]; lipid A biosynthetic process [GO:0009245]
HSIVP1_RS06695	- -	phosphatidate phosphatase	1.90	0.03	0.11	#N/A
HSIVP1_RS05880	Vpar_1227	rod shape-determining protein	1.85	0.03	0.11	integral component of membrane [GO:0016021]; regulation of cell shape [GO:0008360]
HSIVP1_RS06165	Vpar_1283	dyp-type peroxidase family protein	-1.17	0.03	0.11	heme binding [GO:0020037]; peroxidase activity [GO:0004601]
HSIVP1_RS07975	Vpar_0398	family transcriptional regulator	-4.59	0.03	0.11	DNA-binding transcription factor activity [GO:0003700]
HSIVP1_RS06135	glnS	glutamine--trna ligase	-1.25	0.03	0.11	cytoplasm [GO:0005737]; ATP binding [GO:0005524]; glutamine-tRNA ligase activity [GO:0004819]; glutaminyl-tRNA aminoacylation [GO:0006425]; glutamyl-tRNA aminoacylation [GO:0006424]
HSIVP1_RS02270	Vpar_1072	membrane protein	1.27	0.03	0.11	integral component of membrane [GO:0016021]
HSIVP1_RS05645	- -	hydrogenase expression formation protein	1.69	0.03	0.11	enzyme activator activity [GO:0008047]; peptidase activity [GO:0008233]
HSIVP1_RS08970	- -	iron-sulfur cluster repair di-iron protein	-4.58	0.03	0.11	cytoplasm [GO:0005737]; metal ion binding [GO:0046872]
HSIVP1_RS06970	ilvD	dihydroxy-acid dehydratase	-1.97	0.03	0.11	4 iron, 4 sulfur cluster binding [GO:0051539]; dihydroxy-acid dehydratase activity [GO:0004160]; metal ion binding [GO:0046872]; isoleucine biosynthetic process [GO:0009097]; valine biosynthetic process [GO:0009099]
HSIVP1_RS07640	- -	pf12889 family protein	1.19	0.03	0.11	integral component of membrane [GO:0016021]
HSIVP1_RS05870	cobQ	cobyric acid synthase	1.27	0.03	0.11	0
HSIVP1_RS08170	- -	serine threonine protein phosphatase	-1.41	0.03	0.11	hydrolase activity [GO:0016787]
HSIVP1_RS00575	Vpar_0141	cysteine synthase	1.85	0.03	0.11	cysteine synthase activity [GO:0004124]; cysteine biosynthetic process from serine [GO:0006535]
HSIVP1_RS05900	- -	septum formation protein maf	3.65	0.03	0.11	cytoplasm [GO:0005737]; nucleoside-triphosphate diphosphatase activity [GO:0047429]

GeneSymbol (NCBI)	StringDB_locus tags	Gene description	$Log_2FoldChange^a$	*P* value	P_{adj}^b	GO terms
HSIVP1_RS07845	- -	methionine abc transporter substrate-binding protein	-4.59	0.03	0.11	0
HSIVP1_RS00850	Vpar_0400	transcriptional family	1.96	0.03	0.11	DNA binding [GO:0003677]; DNA-binding transcription factor activity [GO:0003700]; transcription, DNA-templated [GO:0006351]
HSIVP1_RS05285	murC	udp-n-acetylmuramate--l-alanine ligase	1.43	0.03	0.11	cytoplasm [GO:0005737]; ATP binding [GO:0005524]; UDP-N-acetylmuramate-L-alanine ligase activity [GO:0008763]; cell cycle [GO:0007049]; cell division [GO:0051301]; cell wall organization [GO:0071555]; peptidoglycan biosynthetic process [GO:0009252]; regulation of cell shape [GO:0008360]
HSIVP1_RS01685	- -	periplasmic pf09992 family	1.24	0.03	0.11	0
HSIVP1_RS01405	Vpar_0325	cyclopropane-fatty-acyl-phospholipid synthase	-2.00	0.03	0.11	cyclopropane-fatty-acyl-phospholipid synthase activity [GO:0008825]; lipid biosynthetic process [GO:0008610]
HSIVP1_RS07350	rplJ	50s ribosomal protein l10	-1.65	0.03	0.11	ribosome [GO:0005840]; large ribosomal subunit rRNA binding [GO:0070180]; ribosome biogenesis [GO:0042254]; translation [GO:0006412]
HSIVP1_RS02800	- -	bifunctional n-acetylglucosamine-1-phosphate uridyltransferase glucosamine-1-phosphate acetyltransferase	1.58	0.03	0.12	cytoplasm [GO:0005737]; glucosamine-1-phosphate N-acetyltransferase activity [GO:0019134]; magnesium ion binding [GO:0000287]; UDP-N-acetylglucosamine diphosphorylase activity [GO:0003977]; cell morphogenesis [GO:0000902]; cell wall organization [GO:0071555]; lipid A biosynthetic process [GO:0009245]; lipopolysaccharide biosynthetic process [GO:0009103]; peptidoglycan biosynthetic process [GO:0009252]; regulation of cell shape [GO:0008360]; UDP-N-acetylglucosamine biosynthetic process [GO:0006048]
HSIVP1_RS06940	- -	pf11694 family protein	1.27	0.03	0.12	integral component of membrane [GO:0016021]
HSIVP1_RS01120	metAA	homoserine o-succinyltransferase	1.61	0.03	0.12	cytoplasm [GO:0005737]; homoserine O-acetyltransferase activity [GO:0004414]; homoserine O-succinyltransferase activity [GO:0008899]; L-methionine biosynthetic process from homoserine via O-succinyl-L-homoserine and cystathionine [GO:0019281]
HSIVP1_RS10055	- -	n-acetylglucosaminylphosphatidylinositol deacetylase	1.15	0.03	0.12	0
HSIVP1_RS06025	Vpar_1324	gtp-binding protein	1.36	0.03	0.12	GTP binding [GO:0005525]; metal ion binding [GO:0046872]; cell septum assembly [GO:0090529]
HSIVP1_RS07325	rpoB	dna-directed rna polymerase subunit beta	1.22	0.03	0.12	DNA binding [GO:0003677]; DNA-directed 5'-3' RNA polymerase activity [GO:0003899]; transcription, DNA-templated [GO:0006351]
HSIVP1_RS01925	rph	ribonuclease ph	-3.01	0.03	0.12	3'-5'-exoribonuclease activity [GO:0000175]; tRNA binding [GO:0000049]; tRNA nucleotidyltransferase activity [GO:0009022]; rRNA catabolic process [GO:0016075]; rRNA processing [GO:0006364]; tRNA processing [GO:0008033]
HSIVP1_RS03740	Vpar_0810	s41 family	1.63	0.03	0.12	serine-type peptidase activity [GO:0008236]

GeneSymbol (NCBI)	StringDB_locus tags	Gene description	$Log_2FoldChange^a$	*P* value	P_{adj}^b	GO terms
HSIVP1_RS00565	queC	preq synthase	-2.53	0.03	0.12	cytoplasm [GO:0005737]; oxidoreductase activity, acting on other nitrogenous compounds as donors, with NAD or NADP as acceptor [GO:0046857]; preQ1 synthase activity [GO:0033739]; queuosine biosynthetic process [GO:0008616]
HSIVP1_RS09300	Vpar_1072	membrane protein	-1.19	0.03	0.12	integral component of membrane [GO:0016021]
HSIVP1_RS02710	ruvA	holliday junction dna helicase	1.45	0.03	0.12	ATP binding [GO:0005524]; DNA binding [GO:0003677]; four-way junction helicase activity [GO:0009378]; DNA recombination [GO:0006310]; DNA repair [GO:0006281]; SOS response [GO:0009432]
HSIVP1_RS01820	Vpar_1072	membrane protein	1.24	0.03	0.12	0
HSIVP1_RS03550	- -	dutp diphosphatase	1.26	0.03	0.12	dUTP diphosphatase activity [GO:0004170]; metal ion binding [GO:0046872]; dUTP metabolic process [GO:0046080]
HSIVP1_RS09630	Vpar_0020	o-antigen polymerase	1.42	0.03	0.12	integral component of membrane [GO:0016021]
HSIVP1_RS02390	Vpar_0554	peptidase	1.22	0.03	0.12	0
HSIVP1_RS07165	map	methionine aminopeptidase	1.14	0.03	0.12	metal ion binding [GO:0046872]; metalloaminopeptidase activity [GO:0070006]; protein initiator methionine removal [GO:0070084]
HSIVP1_RS01180	- -	small conductance mechanosensitive ion channel family protein	-1.60	0.03	0.12	integral component of membrane [GO:0016021]; transmembrane transport [GO:0055085]
HSIVP1_RS09755	Vpar_0993	conserved domain protein	1.17	0.03	0.13	0
HSIVP1_RS05890	Vpar_1227	rod shape-determining protein	1.80	0.03	0.13	cell morphogenesis [GO:0000902]
HSIVP1_RS02895	ksgA	dimethyladenosine transferase	1.22	0.03	0.13	cytoplasm [GO:0005737]; 16S rRNA (adenine(1518)-N(6)/adenine(1519)-N(6))-dimethyltransferase activity [GO:0052908]; RNA binding [GO:0003723]; rRNA (adenine-N6,N6-)-dimethyltransferase activity [GO:0000179]
HSIVP1_RS03670	- -	glutamine abc transporter permease	1.19	0.04	0.13	ATP-binding cassette (ABC) transporter complex [GO:0043190]; transmembrane transporter activity [GO:0022857]; nitrogen compound transport [GO:0071705]
HSIVP1_RS04070	mutL	dna mismatch repair protein	1.17	0.04	0.13	ATP binding [GO:0005524]; damaged DNA binding [GO:0003684]; mismatched DNA binding [GO:0030983]; mismatch repair [GO:0006298]
HSIVP1_RS07885	Vpar_0011	membrane-fusion protein	1.16	0.04	0.13	0
HSIVP1_RS02290	greA	transcription elongation factor	1.25	0.04	0.13	DNA binding [GO:0003677]; RNA polymerase binding [GO:0070063]; translation elongation factor activity [GO:0003746]; regulation of DNA-templated transcription, elongation [GO:0032784]
HSIVP1_RS02770	Vpar_0554	peptidase	-1.61	0.04	0.13	#N/A
HSIVP1_RS07725	Vpar_0603	sodium:proton antiporter	1.39	0.04	0.13	integral component of membrane [GO:0016021]
HSIVP1_RS02170	- -	peptide abc transporter atp-binding protein	1.24	0.04	0.13	ATP binding [GO:0005524]; ATPase activity [GO:0016887]; peptide transport [GO:0015833]
HSIVP1_RS08050	- -	pf09924 family protein	-1.52	0.04	0.13	0
HSIVP1_RS08430	Vpar_0006	hypothetical protein	2.00	0.04	0.13	0

GeneSymbol (NCBI)	StringDB_locus tags	Gene description	$Log_2FoldChange^a$	*P* value	P_{adj}^b	GO terms
HSIVP1_RS01605	menD	2-succinyl-5-enolpyruvyl-6-hydroxy-3-cyclohexene-1-carboxylate synthase	1.23	0.04	0.14	2-succinyl-5-enolpyruvyl-6-hydroxy-3-cyclohexene-1-carboxylic-acid synthase activity [GO:0070204]; magnesium ion binding [GO:0000287]; manganese ion binding [GO:0030145]; thiamine pyrophosphate binding [GO:0030976]; menaquinone biosynthetic process [GO:0009234]
HSIVP1_RS02920	Vpar_1707	domain protein	1.14	0.04	0.14	transition metal ion binding [GO:0046914]
HSIVP1_RS08375	Vpar_0006	hypothetical protein	1.30	0.04	0.14	#N/A
HSIVP1_RS07435	hypA	hydrogenase nickel insertion protein	-1.68	0.04	0.14	nickel cation binding [GO:0016151]; zinc ion binding [GO:0008270]; cellular protein modification process [GO:0006464]; protein maturation [GO:0051604]
HSIVP1_RS07035	Vpar_0400	transcriptional family	1.09	0.04	0.14	#N/A
HSIVP1_RS06190	Vpar_1264	xanthine permease	-1.80	0.04	0.14	integral component of membrane [GO:0016021]; transmembrane transporter activity [GO:0022857]
HSIVP1_RS06110	Vpar_1272	diaminopropionate ammonia-lyase	-1.08	0.04	0.14	diaminopropionate ammonia-lyase activity [GO:0008838]; L-threonine ammonia-lyase activity [GO:0004794]; pyridoxal phosphate binding [GO:0030170]
HSIVP1_RS00425	Vpar_0154	amidase	-1.35	0.04	0.14	isochorismatase activity [GO:0008908]
HSIVP1_RS06445	Vpar_1386	family domain protein	3.93	0.04	0.14	integral component of membrane [GO:0016021]
HSIVP1_RS03605	- -	transcription termination factor	-2.60	0.04	0.14	cytoplasm [GO:0005737]; DNA-binding transcription factor activity [GO:0003700]; RNA binding [GO:0003723]; DNA-templated transcription, termination [GO:0006353]; transcription antitermination [GO:0031564]
HSIVP1_RS08930	- -	two-component sensor histidine kinase	1.17	0.04	0.14	integral component of membrane [GO:0016021]; intracellular [GO:0005622]; ATP binding [GO:0005524]; phosphorelay sensor kinase activity [GO:0000155]
HSIVP1_RS02595	Vpar_0400	transcriptional family	1.16	0.04	0.14	DNA binding [GO:0003677]; DNA-binding transcription factor activity [GO:0003700]; transcription, DNA-templated [GO:0006351]
HSIVP1_RS04765	Vpar_1011	rna polymerase sigma-h factor	1.25	0.04	0.15	DNA binding [GO:0003677]; DNA-binding transcription factor activity [GO:0003700]; sigma factor activity [GO:0016987]; DNA-templated transcription, initiation [GO:0006352]
HSIVP1_RS00695	Vpar_1072	membrane protein	-1.70	0.04	0.15	integral component of membrane [GO:0016021]
HSIVP1_RS02325	serS	serine--trna ligase	1.11	0.04	0.15	cytoplasm [GO:0005737]; ATP binding [GO:0005524]; serine-tRNA ligase activity [GO:0004828]; selenocysteine biosynthetic process [GO:0016260]; selenocysteinyl-tRNA(Sec) biosynthetic process [GO:0097056]; seryl-tRNA aminoacylation [GO:0006434]
HSIVP1_RS02930	Vpar_0006	hypothetical protein	1.21	0.04	0.15	0
HSIVP1_RS08800	rplI	ribosomal protein l9	1.09	0.04	0.15	ribosome [GO:0005840]; rRNA binding [GO:0019843]; structural constituent of ribosome [GO:0003735]; translation [GO:0006412]
HSIVP1_RS09250	Vpar_1730	carboxymuconolactone decarboxylase	-1.44	0.04	0.15	4-carboxymuconolactone decarboxylase activity [GO:0047575]; peroxiredoxin activity [GO:0051920]

GeneSymbol (NCBI)	StringDB_locus tags	Gene description	$Log_2FoldChange$[a]	*P* value	P_{adj}[b]	GO terms
HSIVP1_RS00920	- -	Respiratory nitrate reductase alpha chain	-1.07	0.04	0.15	nitrate reductase complex [GO:0009325]; 4 iron, 4 sulfur cluster binding [GO:0051539]; metal ion binding [GO:0046872]; molybdopterin cofactor binding [GO:0043546]; nitrate reductase activity [GO:0008940]; nitrate metabolic process [GO:0042126]
HSIVP1_RS04915	- -	trna threonylcarbamoyladenosine biosynthesis protein	-1.52	0.04	0.15	peptidase activity [GO:0008233]; tRNA threonylcarbamoyladenosine modification [GO:0002949]
HSIVP1_RS09455	- -	pf14584 family protein	1.12	0.04	0.15	integral component of membrane [GO:0016021]
HSIVP1_RS02795	Vpar_0606	pur operon repressor	2.20	0.05	0.15	adenine phosphoribosyltransferase activity [GO:0003999]; DNA binding [GO:0003677]; negative regulation of purine nucleobase metabolic process [GO:0045982]; negative regulation of transcription, DNA-templated [GO:0045892]; nucleoside metabolic process [GO:0009116]
HSIVP1_RS04975	- -	cdp-diacylglycerol-serine o-phosphatidyltransferase	1.19	0.05	0.15	integral component of membrane [GO:0016021]; CDP-diacylglycerol-serine O-phosphatidyltransferase activity [GO:0003882]; phospholipid biosynthetic process [GO:0008654]
HSIVP1_RS09615	- -	arylsulfatase	1.09	0.05	0.16	integral component of membrane [GO:0016021]; sulfuric ester hydrolase activity [GO:0008484]
HSIVP1_RS02750	Vpar_0387	mfs transporter	1.44	0.05	0.16	integral component of membrane [GO:0016021]; transmembrane transport [GO:0055085]
HSIVP1_RS09555	gyrB	dna gyrase subunit b	-1.09	0.05	0.16	chromosome [GO:0005694]; cytoplasm [GO:0005737]; ATP binding [GO:0005524]; DNA binding [GO:0003677]; DNA topoisomerase type II (ATP-hydrolyzing) activity [GO:0003918]; metal ion binding [GO:0046872]; DNA topological change [GO:0006265]; DNA-dependent DNA replication [GO:0006261]
HSIVP1_RS06420	- -	potassium uptake system protein	-1.58	0.05	0.16	cation transmembrane transporter activity [GO:0008324]; potassium ion transport [GO:0006813]
HSIVP1_RS05600	Vpar_1176	o-methyltransferase	1.24	0.05	0.16	O-methyltransferase activity [GO:0008171]
HSIVP1_RS02395	- -	outer membrane omp85 family	-1.07	0.05	0.16	outer membrane [GO:0019867]
HSIVP1_RS03915	- -	pf04025 domain protein	1.92	0.05	0.16	0
HSIVP1_RS05845	Vpar_1072	membrane protein	-1.37	0.05	0.16	#N/A
HSIVP1_RS05340	- -	glutamyl-queuosine trna synthetase	-2.32	0.05	0.16	cytoplasm [GO:0005737]; aminoacyl-tRNA ligase activity [GO:0004812]; ATP binding [GO:0005524]; tRNA aminoacylation for protein translation [GO:0006418]

[a]Log2 Fold Change in Coaggregates versus monocultures. Genes down-regulated in coaggregates have negative numbers.

[b]Blue shading indicates genes where $P_{adj} > 0.05$ and $P < 0.05$

Table S3. Genes regulated in *S. gordonii* following coaggregation with *V. parvula* ($P < 0.05$).

GeneSymbol (NCBI)	StringDB_locus tags	Gene Description	$Log_2FoldChange^a$	P value	P_{adj}^b	GO terms
SGO_RS01315	SGO_0267	transporter	-1.13	0.00	0.85	integral component of membrane [GO:0016021]; amino acid transmembrane transporter activity [GO:0015171]
SGO_RS06225	SGO_RS06225	thioredoxin signature protein	1.80	0.00	0.85	-
SGO_RS06800	sufB-2 SGO_1387	Fe-S cluster assembly protein	-1.08	0.00	0.85	iron-sulfur cluster assembly [GO:0016226]
SGO_RS03505	dgkA SGO_0712	diacylglycerol kinase	-1.44	0.00	0.85	integral component of membrane [GO:0016021]; diacylglycerol kinase activity [GO:0004143]; phospholipid biosynthetic process [GO:0008654]
SGO_RS00210	SGO_0044	PTS system sorbose subfamily IIB component	-1.05	0.00	0.85	cytoplasm [GO:0005737]; D-glucosamine PTS permease activity [GO:0103111]; protein-N(PI)-phosphohistidine-sugar phosphotransferase activity [GO:0008982]; phosphoenolpyruvate-dependent sugar phosphotransferase system [GO:0009401]
SGO_RS09100	SGO_1858	sucrose-6-phosphate hydrolase	-1.33	0.00	0.85	cytoplasm [GO:0005737]; sucrose alpha-glucosidase activity [GO:0004575]; carbohydrate metabolic process [GO:0005975]
SGO_RS03500	ybeY SGO_0711	metalloprotease	-1.08	0.00	0.85	cytoplasm [GO:0005737]; endonuclease activity [GO:0004519]; metal ion binding [GO:0046872]; metalloendopeptidase activity [GO:0004222]; rRNA processing [GO:0006364]
SGO_RS09095	SGO_1857	PTS beta-glucoside transporter subunit EIIBCA	-1.05	0.00	0.85	integral component of membrane [GO:0016021]; plasma membrane [GO:0005886]; D-glucosamine PTS permease activity [GO:0103111]; kinase activity [GO:0016301]; protein-N(PI)-phosphohistidine-sugar phosphotransferase activity [GO:0008982]; phosphoenolpyruvate-dependent sugar phosphotransferase system [GO:0009401]
SGO_RS01065	mntH SGO_0217	manganese transporter	-1.07	0.00	0.85	integral component of membrane [GO:0016021]; plasma membrane [GO:0005886]; metal ion binding [GO:0046872]; metal ion transmembrane transporter activity [GO:0046873]; symporter activity [GO:0015293]
SGO_RS02580	SGO_0522	acylphosphatase	-1.15	0.00	0.85	acylphosphatase activity [GO:0003998]
SGO_RS03395	SGO_0689	pf08796 family protein	1.60	0.00	0.85	-
SGO_RS01450	SGO_RS01450	transcriptional regulator	1.42	0.01	0.85	-
SGO_RS05750	SGO_RS05750	thiamine biosynthesis protein	-1.03	0.01	0.85	-
SGO_RS01930	SGO_0387	dihydrodipicolinate reductase	1.54	0.01	0.96	4-hydroxy-tetrahydrodipicolinate reductase [GO:0008839]; lysine biosynthetic process via diaminopimelate [GO:0009089]
SGO_RS02125	SGO_0427	universal stress protein	-1.04	0.01	0.96	cytoplasm [GO:0005737]; response to stress [GO:0006950]
SGO_RS10165	SGO_2076	integrase	-0.98	0.01	1.00	DNA binding [GO:0003677]; DNA integration [GO:0015074]; DNA recombination [GO:0006310]
SGO_RS01070	SGO_RS01070	partial	-0.96	0.01	1.00	-

GeneSymbol (NCBI)	StringDB_locus tags	Gene Description	$Log_2FoldChange^a$	*P* value	P_{adj}^{b}	GO terms
SGO_RS01740	SGO_0352	sugar ABC transporter ATP-binding protein	-0.91	0.01	1.00	ATP-binding cassette (ABC) transporter complex [GO:0043190]; ATP binding [GO:0005524]; ATPase activity [GO:0016887]; transporter activity [GO:0005215]; transport [GO:0006810]
SGO_RS07170	SGO_1461	domain protein	1.48	0.01	1.00	integral component of membrane [GO:0016021]
SGO_RS00365	SGO_0075	hypothetical protein	1.20	0.01	1.00	integral component of membrane [GO:0016021]
SGO_RS02770	SGO_0561	toxin	-0.96	0.01	1.00	-
SGO_RS00215	SGO_0045	PTS fructose transporter subunit IIC	-0.91	0.01	1.00	integral component of membrane [GO:0016021]; D-glucosamine PTS permease activity [GO:0103111]; protein-N(PI)-phosphohistidine-sugar phosphotransferase activity [GO:0008982]; phosphoenolpyruvate-dependent sugar phosphotransferase system [GO:0009401]
SGO_RS04230	SGO_0862	hypothetical protein	1.55	0.02	1.00	integral component of membrane [GO:0016021]
SGO_RS03570	SGO_0726	family transcriptional regulator	-1.19	0.02	1.00	DNA binding [GO:0003677]; regulation of transcription, DNA-templated [GO:0006355]; transcription, DNA-templated [GO:0006351]
SGO_RS03555	tehB SGO_0722	tellurite resistance protein	1.34	0.02	1.00	cytoplasm [GO:0005737]; S-adenosylmethionine-dependent methyltransferase activity [GO:0008757]; response to tellurium ion [GO:0046690]
SGO_RS02790	sgc SGO_0566	serine protease	0.88	0.02	1.00	cell wall [GO:0005618]; extracellular region [GO:0005576]; membrane [GO:0016020]; serine-type endopeptidase activity [GO:0004252]; pathogenesis [GO:0009405]
SGO_RS03370	SGO_0684	pf08860 domain protein	-0.86	0.02	1.00	-
SGO_RS04845	SGO_0988	transcriptional regulator	0.93	0.02	1.00	intracellular [GO:0005622]; DNA binding [GO:0003677]; DNA binding transcription factor activity [GO:0003700]; transcription, DNA-templated [GO:0006351]
SGO_RS10185	SGO_2080	xre family transcriptional regulator	-0.96	0.02	1.00	sequence-specific DNA binding [GO:0043565]
SGO_RS02590	SGO_0524	HD domain protein	-0.83	0.02	1.00	-
SGO_RS01075	SGO_RS01075	transposase	-0.87	0.02	1.00	-
SGO_RS00985	SGO_0201	3 -5 exonuclease	0.86	0.02	1.00	-
SGO_RS09785	SGO_RS09785	membrane protein	-0.82	0.02	1.00	-
SGO_RS07880	SGO_RS07880	transcriptional family	-0.86	0.02	1.00	-
SGO_RS10300	abpA SGO_2105	amylase-binding protein	-0.82	0.02	1.00	-
SGO_RS08385	amiD SGO_1710	peptide abc transporter permease	0.90	0.02	1.00	integral component of membrane [GO:0016021]; plasma membrane [GO:0005886]; transport [GO:0006810]
SGO_RS01335	SGO_0271	Zn-dependent protease	1.00	0.02	1.00	-
SGO_RS06260	mscL SGO_1275	large conductance mechanosensitive channel protein	1.01	0.02	1.00	integral component of plasma membrane [GO:0005887]; mechanosensitive ion channel activity [GO:0008381]
SGO_RS04880	SGO_0996	short-chain dehydrogenase	-0.81	0.03	1.00	oxidoreductase activity [GO:0016491]

GeneSymbol (NCBI)	StringDB_locus tags	Gene Description	$Log_2FoldChange^a$	*P* value	P_{adj}^{b}	GO terms
SGO_RS07435	lacC SGO_1517	tagatose-6-phosphate kinase	-0.79	0.03	1.00	ATP binding [GO:0005524]; tagatose-6-phosphate kinase activity [GO:0009024]; D-tagatose 6-phosphate catabolic process [GO:2001059]; lactose catabolic process via tagatose-6-phosphate [GO:0019512]
SGO_RS02020	SGO_0405	beta-n-acetylhexosaminidase	0.83	0.03	1.00	extracellular region [GO:0005576]; integral component of membrane [GO:0016021]; beta-N-acetylhexosaminidase activity [GO:0004563]; N-acetyl-beta-D-galactosaminidase activity [GO:0102148]; carbohydrate metabolic process [GO:0005975]
SGO_RS03085	recX SGO_0626	recombination regulator	-0.91	0.03	1.00	cytoplasm [GO:0005737]; regulation of DNA repair [GO:0006282]
SGO_RS03795	ccpA SGO_0773	catabolite control protein A	-0.81	0.03	1.00	protein-DNA complex [GO:0032993]; bacterial-type RNA polymerase transcriptional repressor activity, sequence-specific DNA binding [GO:0001217]; transcription regulatory region sequence-specific DNA binding [GO:0000976]; negative regulation of transcription, DNA-templated [GO:0045892]; transcription, DNA-templated [GO:0006351]
SGO_RS00770	SGO_0156	hypothetical protein	0.97	0.03	1.00	integral component of membrane [GO:0016021]
SGO_RS03680	SGO_0749	glutathione-disulfide reductase	0.81	0.03	1.00	cell [GO:0005623]; electron transfer activity [GO:0009055]; flavin adenine dinucleotide binding [GO:0050660]; glutathione-disulfide reductase activity [GO:0004362]; NADP binding [GO:0050661]; cell redox homeostasis [GO:0045454]; glutathione metabolic process [GO:0006749]
SGO_RS05225	SGO_RS05225	hypothetical protein	1.80	0.03	1.00	-
SGO_RS05250	SGO_1069	aminopeptidase N	0.97	0.03	1.00	aminopeptidase activity [GO:0004177]; metallopeptidase activity [GO:0008237]; zinc ion binding [GO:0008270]
SGO_RS08605	scrK SGO_1755	fructokinase	-0.80	0.03	1.00	fructokinase activity [GO:0008865]
SGO_RS07610	glgC SGO_1553	glucose-1-phosphate adenylyltransferase	-0.76	0.03	1.00	ATP binding [GO:0005524]; glucose-1-phosphate adenylyltransferase activity [GO:0008878]; glycogen biosynthetic process [GO:0005978]
SGO_RS05055	SGO_1030	30S ribosomal protein S15	-0.77	0.04	1.00	integral component of membrane [GO:0016021]
SGO_RS05585	SGO_RS05585	esterase	1.91	0.04	1.00	-
SGO_RS03935	thrB SGO_0802	homoserine kinase	0.91	0.04	1.00	cytoplasm [GO:0005737]; ATP binding [GO:0005524]; homoserine kinase activity [GO:0004413]; threonine biosynthetic process [GO:0009088]
SGO_RS03460	SGO_0703	Fur family transcriptional regulator	-0.75	0.04	1.00	DNA binding [GO:0003677]; DNA binding transcription factor activity [GO:0003700]
SGO_RS08800	pflC SGO_1794	pyruvate formate-lyase-activating enzyme	-0.73	0.04	1.00	4 iron, 4 sulfur cluster binding [GO:0051539]; [formate-C-acetyltransferase]-activating enzyme activity [GO:0043365]; transferase activity [GO:0016740]

GeneSymbol (NCBI)	StringDB_locus tags	Gene Description	$Log_2FoldChange^a$	*P* value	P_{adj}^{b}	GO terms
SGO_RS08395	sarA hppA SGO_1712	peptide ABC transporter atp-binding protein	0.78	0.04	1.00	ATP-binding cassette (ABC) transporter complex [GO:0043190]; protein transport [GO:0015031]; transmembrane transport [GO:0055085]
SGO_RS00220	SGO_0046	PTS fructose transporter subunit IID	-0.89	0.04	1.00	integral component of membrane [GO:0016021]; D-glucosamine PTS permease activity [GO:0103111]; protein-N(PI)-phosphohistidine-sugar phosphotransferase activity [GO:0008982]; phosphoenolpyruvate-dependent sugar phosphotransferase system [GO:0009401]
SGO_RS06525	SGO_1330	ABC transporter ATP-binding protein	-0.79	0.04	1.00	integral component of membrane [GO:0016021]; ATP binding [GO:0005524]; ATPase activity, coupled to transmembrane movement of substances [GO:0042626]
SGO_RS01125	SGO_0228	family transcriptional regulator	0.91	0.04	1.00	DNA binding [GO:0003677]; regulation of transcription, DNA-templated [GO:0006355]
SGO_RS03940	SGO_0803	uncharacterised protein	0.82	0.04	1.00	integral component of membrane [GO:0016021]
SGO_RS09155	SGO_1869	hypothetical protein	0.95	0.04	1.00	integral component of membrane [GO:0016021]
SGO_RS01505	SGO_0305	lantibiotic abc transporter permease	-0.89	0.04	1.00	integral component of membrane [GO:0016021]; transport [GO:0006810]
SGO_RS09195	SGO_RS09195	family transcriptional regulator	-1.13	0.04	1.00	-
SGO_RS04645	deaD SGO_0949	RNA helicase	0.75	0.05	1.00	ATP binding [GO:0005524]; helicase activity [GO:0004386]; nucleic acid binding [GO:0003676]
SGO_RS06050	ldh SGO_1232	l-lactate dehydrogenase 1	-0.71	0.05	1.00	cytoplasm [GO:0005737]; L-lactate dehydrogenase activity [GO:0004459]; carbohydrate metabolic process [GO:0005975]; carboxylic acid metabolic process [GO:0019752]
SGO_RS10595	SGO_RS10595	truncated hypothetical protein	0.70	0.05	1.00	-
SGO_RS04160	rpmE rpmE2 SGO_0848	ribosomal protein l31	0.81	0.05	1.00	ribosome [GO:0005840]; structural constituent of ribosome [GO:0003735]; translation [GO:0006412]
SGO_RS01500	SGO_0304	lantibiotic ABC transporter permease	-0.85	0.05	1.00	integral component of membrane [GO:0016021]; transport [GO:0006810]
SGO_RS02775	SGO_0562	DNA-binding protein	-0.73	0.05	1.00	sequence-specific DNA binding [GO:0043565]
SGO_RS08120	SGO_1657	-	-0.81	0.05	1.00	integral component of membrane [GO:0016021]

[a]Log2 Fold Change in Coaggregates versus monocultures. Genes down-regulated in coaggregates have negative numbers.

[b]Blue shading indicates genes where $P_{adj} > 0.05$ and $P < 0.05$. There were no genes that met the criterion $P_{adj} < 0.05$.

Table S4. Genes in the enriched GO category 'oxidoreductase' that were regulated in *V. parvula* following coaggregation with *S. gordonii* (*P* adj < 0.05).

Oxidoreductase	Gene/Vpar ID	Gene description	$Log_2FoldChange^a$	*P* value	P_{adj}
HSIVP1_RS00195	Vpar_0077	FAD-dependent oxidoreductase	-6.56	0.00	0.00
HSIVP1_RS00930	Vpar_0224	nitrate reductase molybdenum cofactor assembly chaperone	-1.67	0.00	0.01
HSIVP1_RS01385	- -	NADH-dependent oxidoreductase	-3.84	0.00	0.00
HSIVP1_RS01410	Vpar_0329	diaminopimelate dehydrogenase	-2.62	0.00	0.00
HSIVP1_RS01455	- -	conserved domain protein	1.64	0.00	0.02
HSIVP1_RS01945	Vpar_1595	malate dehydrogenase	-2.20	0.00	0.00
HSIVP1_RS02075	Vpar_0574	dihydrolipoamide dehydrogenase	-4.54	0.00	0.00
HSIVP1_RS02130	Vpar_0478	cytochrome ubiquinol oxidase subunit i	-2.56	0.00	0.00
HSIVP1_RS02135	Vpar_0478	cytochrome d ubiquinol subunit ii	-4.59	0.00	0.00
HSIVP1_RS02585	- -	dihydropicolinate synthase	1.65	0.00	0.03
HSIVP1_RS03250	Vpar_0690	2-nitropropane dioxygenase	-1.85	0.00	0.00
HSIVP1_RS03260	Vpar_0702	3-oxoacyl-[acyl-carrier-protein] synthase III (*fabH*)	-2.05	0.00	0.00
HSIVP1_RS03405	Vpar_0740	antibiotic biosynthesis monooxygenase	-1.98	0.00	0.01
HSIVP1_RS03885	Vpar_0834	glycerol-3-phosphate dehydrogenase (*gpsA*)	2.88	0.00	0.00
HSIVP1_RS04480	Vpar_0955	pyruvate dehydrogenase	-2.51	0.00	0.00
HSIVP1_RS05330	Vpar_1121	thioredoxin	-1.44	0.01	0.05
HSIVP1_RS05430	Vpar_1143	precorrin-4 c -methyltransferase	-3.14	0.00	0.00
HSIVP1_RS05660	- -	uptake hydrogenase small subunit	-2.23	0.00	0.00
HSIVP1_RS05665	Vpar_1121	thioredoxin	-4.34	0.00	0.00
HSIVP1_RS05740	Vpar_0704	radical SAM protein (*queE*)	2.33	0.00	0.00
HSIVP1_RS05770	- -	coproporphyrinogen dehydrogenase	-1.58	0.00	0.02
HSIVP1_RS06245	Vpar_1301	GMP reductase (*guaC*)	-2.23	0.00	0.02
HSIVP1_RS06715	Vpar_1382	aldehyde dehydrogenase	-2.91	0.00	0.00
HSIVP1_RS06725	Vpar_1384	dihydrofolate reductase	-3.16	0.01	0.03
HSIVP1_RS06885	Vpar_1424	uroporphyrinogen-iii c-methyltransferase	-5.40	0.00	0.00
HSIVP1_RS06910	Vpar_1429	pyrroline-5-carboxylate reductase (*proC*)	-2.20	0.00	0.01

HSIVP1_RS07105	Vpar_1460	2-oxoacid ferredoxin oxidoreductase subunit beta	-2.02	0.00	0.00
HSIVP1_RS07610	Vpar_1550	glutamate dehydrogenase	-5.72	0.00	0.00
HSIVP1_RS07820	Vpar_1594	molybdopterin oxidoreductase	-3.76	0.00	0.00
HSIVP1_RS07895	Vpar_1606	ribonucleoside-diphosphate reductase beta subunit	-3.95	0.00	0.00
HSIVP1_RS08035	Vpar_1637	glutamate synthase	-4.92	0.00	0.00
HSIVP1_RS08910	Vpar_1703	heterodisulfide reductase subunit b	-9.71	0.00	0.00
HSIVP1_RS08915	Vpar_1704	succinate dehydrogenase and fumarate reductase iron-sulfur protein	-3.50	0.00	0.02
HSIVP1_RS08955	Vpar_0690	dioxygenase	-3.73	0.00	0.00
HSIVP1_RS08995	- -	glutamate synthase homotetrameric	-2.86	0.00	0.00
HSIVP1_RS09010	Vpar_1722	superoxide dismutase	-11.29	0.00	0.00
HSIVP1_RS09230	Vpar_1772	lactaldehyde reductase	-4.59	0.00	0.00
HSIVP1_RS09425	Vpar_0321	nitroreductase	-6.78	0.00	0.00
HSIVP1_RS09580	- -	membrane pf01595 family	-1.81	0.00	0.02

[a]Log2 Fold Change in Coaggregates versus monocultures. Genes down-regulated in coaggregates have negative numbers.

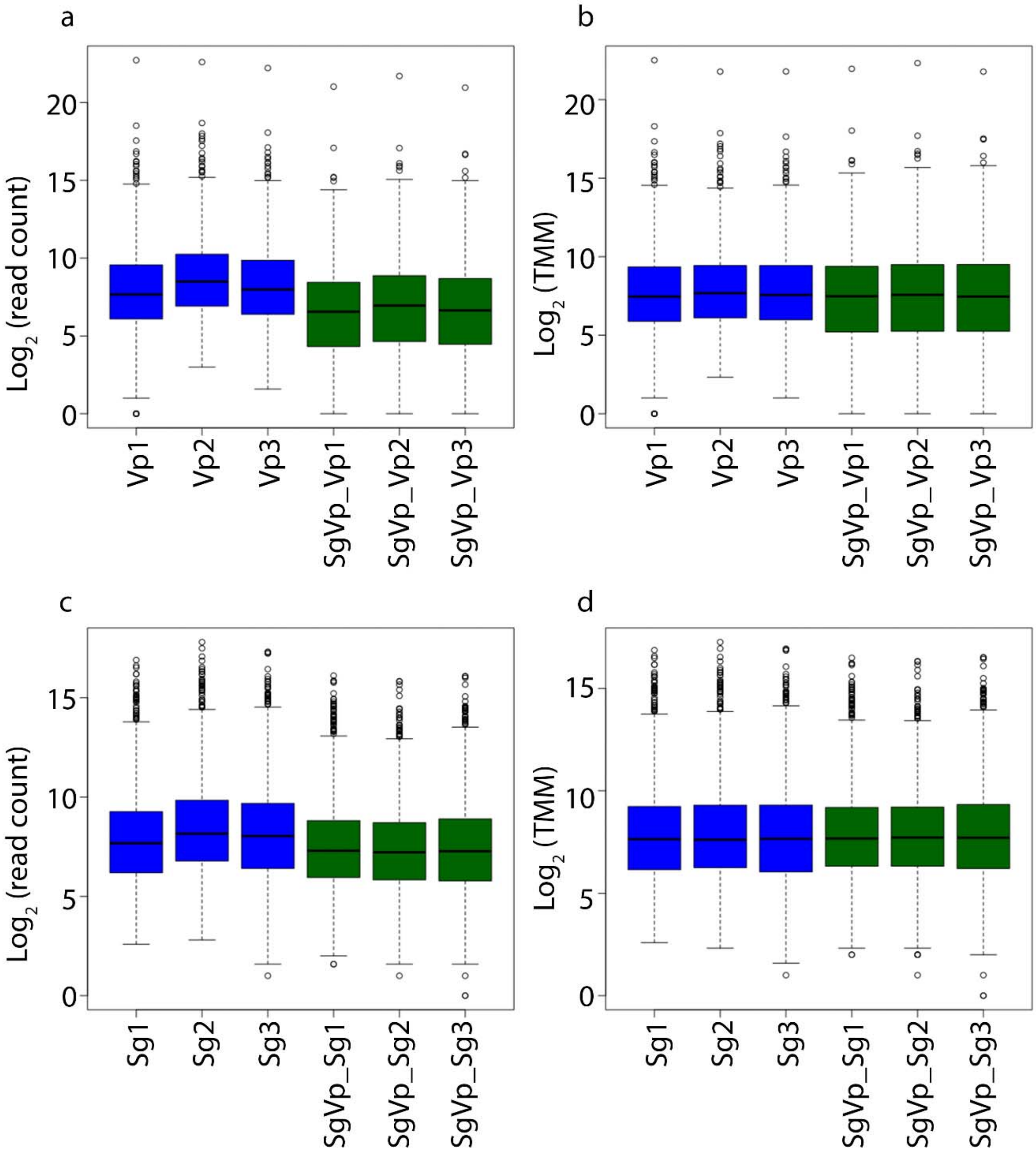

Figure S1. Box plots showing transcript abundance before and after normalization. Gene expression levels *of V. parvula* (a,b) *or S. gordonii* (c,d) genes in coaggregate or monoculture samples are shown before (a,c) or after (b,d) TMM normalization. Median counts are represented as thick black lines, boxes show interquartile ranges and whiskers indicate two SDs from the median. Outliers are indicated by 'o' symbols. In the box plot display, a box is formed with sides at the 25th and 75th percentiles of distribution. A line is also drawn within the box at the level of median. Whiskers are also drawn extending beyond each end of the box with points beyond the whiskers typically indicating outliers.

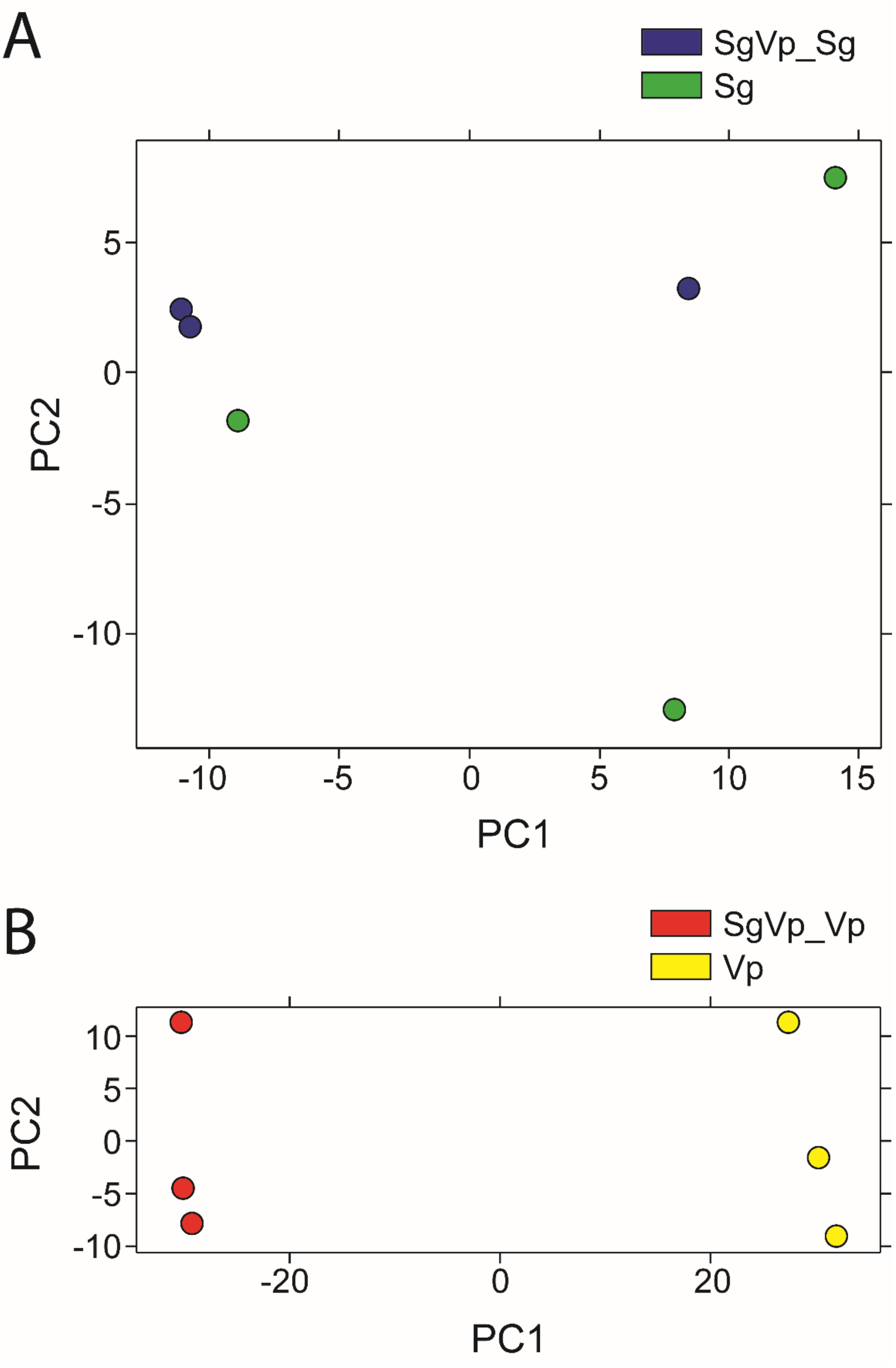

Figure S2. Principal component analysis of biological repeats for monoculture and coaggregation samples. (A) Analysis of *S. gordonii* monoculture samples (Sg) and *S. gordonii* transcripts in coaggregate samples (SgVp_Sg). There was extensive variation between the monoculture samples in particular. (B) *V. parvula* monoculture samples (Vp) clustered separately from *V. parvula* transcripts in coaggregate samples (SgVp_Vp).

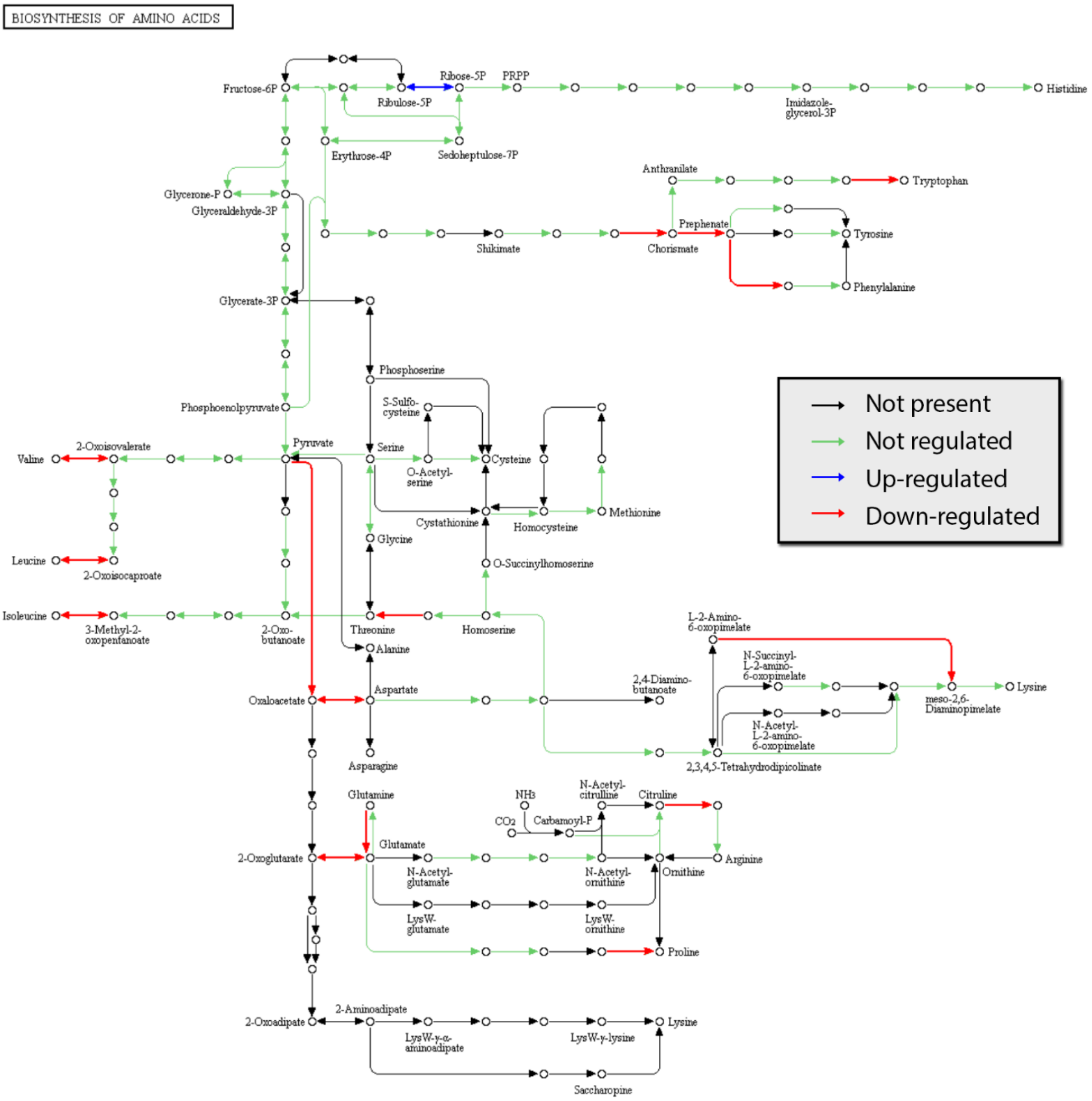

Figure S3. Overview of the KEGG pathway 'Biosynthesis of amino acids', showing genes that were up-regulated or down-regulated in *V. parvula* following coaggregation with *S. gordonii*. A single up-regulated gene is indicated by a blue arrow. Multiple genes encoding steps in biosynthesis of several different amino acids were down-regulated following coaggregation (red arrows). The image was reproduced with permission from Kanehesa laboratories[1].

Reference

1 Kanehisa, M., Furumichi, M., Tanabe, M., Sato, Y. & Morishima, K. KEGG: new perspectives on genomes, pathways, diseases and drugs. *Nucleic Acids Res.* **45**, D353-d361 (2017).
